# An Overview of Spirits Made from Sugarcane Juice

**DOI:** 10.3390/molecules28196810

**Published:** 2023-09-26

**Authors:** Claudine Corbion, Juliette Smith-Ravin, Odile Marcelin, Jalloul Bouajila

**Affiliations:** 1 Laboratoire de Génie Chimique, Université de Toulouse, CNRS-INPT-UPS, 31062 Toulouse, France; claudine.corbion@univ-tlse3.fr; 2 Groupe BIOSPHERES, Campus de Schoelcher, 97275 Schoelcher, Martinique, France; ravinemilie@hotmail.com (J.S.-R.); odile.marcelin@me.com (O.M.)

**Keywords:** fermentation congeners, aging markers, sensory profile, rum, cachaça, rhum agricole

## Abstract

Among the family of sugarcane spirits, those made from juice are diverse and often produced in a traditional way. They must be distinguished from other sugarcane spirits, which are more widely produced and made from other sugarcane derivatives, such as molasses. These alcoholic beverages contribute significantly to the socio-economic development of many countries. However, despite ancestral know-how, there is a lack of contemporary data required to characterize some sugarcane juice spirits (SCJSs) and to overcome the current and future threats that producers will have to face. While preserving their authenticity and specificity, SCJS producers expect to improve and ensure sufficient yield and a superior quality product. Even if the scientific knowledge on these spirits is not comparable, the available data could help identify the critical points to be improved in the making process. This review aims to present the main SCJSs encountered worldwide, defining their specific features through some important aspects with, notably, references to the complex notion of terroir. To continue, we discuss the main steps of the SCJS process from harvesting to aging. Finally, we expose an inventory of SCJS’s chemical compositions and of their sensory description that define the specific organoleptic properties of these spirits.

## 1. Introduction

The term ‘spirit’ refers to any alcoholic beverage made by the fermentation and distillation of cereals, fruits or their sub-products. The history of spirits is related to the development of distillation techniques throughout the centuries. Originally used during ancient times by Persians, Egyptians and Sumerians to obtain perfume and essential oils, this technique was used during the Middle Ages to produce alcohol, called *aqua vitae,* employed in both the medicine and the pharmaceutical sectors to fight against diseases [1]. As a result of the development and industrialization of distillation techniques during the 19th century, the production of spirits expanded across the world with the raw materials that were available [1]. Therefore, the world of spirits is very large and includes baijiu, brandies, rum, shōchū, tequila, vodka and whiskies (Table 1). According to the European Union (EU) regulation 2019/787, a spirit is an alcoholic beverage that possesses particular organoleptic qualities. It is obtained after the distillation of agricultural raw material with a minimum alcohol content of 15% alcohol by volume (ABV) [2]. In 2021, the spirit market worldwide reached 473.6 billion USD and it is expected to increase annually by almost 5% over the next five years [3]. The world’s top spirits are made from fermented cereals or grains such as baijiu from China. The grape-based spirit category is mainly found in European countries, whereas those made from sugarcane and its derivatives (juice, molasses, honey or syrup) are produced in tropical areas (Table 1).

Many countries worldwide have established quality schemes to protect the consumer’s interest and to promote their local and traditional products. In the EU, protected geographical indication helps producers to fight against counterfeiting and ensure that the consumer knows the true origin of the product. In each country, competent authorities control those products comply with quality standards. In France, a Protected Designation of Origin (PDO) called AOC, which stands for ‘Appellation d’Origine Contrôlée’ goes further in the protection of spirits by highlighting the know-how and the specific characteristics linked to the agricultural properties (terroir) of products. The only spirit made from sugarcane juice in a French overseas department with an AOC is the agricultural rum, called ‘Rhum Agricole’, from Martinique [4,5].

The alcoholic beverages discussed in this review are spirits made from fresh sugarcane juice (SCJS). These spirits need to be distinguished from other sugarcane spirits more widely produced, which are made from other sugarcane derivatives, such as molasses [6]. The objective is to gather the information currently available on SCJS and to define their chemical and sensory profiles.

This review deals with the sugarcane, which is the first element to ensure SCJS’s quality. Then, the notion of terroir will be discussed to understand the characteristics that explain the differences or similarities between the SCJSs presented here through their manufacturing process and traditional practices and uses. Finally, the last section will emphasize the chemical and sensorial characteristics of SCJS. A better knowledge of the chemical composition and organoleptic properties of the spirit is essential to promote continuous quality improvement. This strategy has been used for several decades by some producers. As was the case for Rhum Agricole from Martinique during the 1990s, recent studies conducted on cachaça have helped to characterize the profile of this Brazilian SCJS [7,8]. Improving the quality and yield of SCJSs is an economic development strategy that has led to the growth of ‘spiritourism’ [9,10]. Using the current information available in the literature can help producers respond to contemporary challenges (‘drink less but better’ and other environmental and social issues) and promote their traditional products. 

An extensive literature is currently available on cachaça. It could serve as a basis for studying the chemical and sensory characterization of other SCJSs.

## 2. From the Raw Material to the Spirit

The geographical origin of sugarcane (*Saccharum* spp.) is still unclear [11]. A current model suggests that modern sugarcane came from the New Guinea area around 8000 BP and was spread to India and China where the respective cultivars *S. barberi* and *S. sinense* were engendered by the hybridization of *S. officinarum* with *S. spontaneum* [12,13,14] (Table 2). However, a new model indicates the existence of a new cryptic species called *S. cultum*, around 650,000 BP in East Asia, which could be the ancestor of modern cultivars [15].

Human activities led to the migration of these sugarcane varieties across the Pacific and Asian continent around 6000 BP [19], reaching the West around 2400 BP [15]. The crop was introduced to the island of Madeira around 1450 [20], reached the American continent after the second voyage of Christopher Columbus in Santo Domingo [21] and was introduced to the other regions of America such as Brazil, Guadeloupe and Martinique, respectively, in 1520, 1645 and 1650 [20]. It was introduced recently in the 17th century to the Reunion and Mauritius islands and in the 18–19th century to South Africa and Australia [19].

Nowadays, sugarcane is still an important food crop in tropical and subtropical climatic zones such as Central and South America, the Caribbean, India, Africa and Asia-Pacific. In 2021, this crop was cultivated by nearly 100 countries and almost 2 billion tons of cane were harvested. The top 3 producers were Brazil (715 million t), India (405 million t) and China (213 million t) [22]. 

### 2.1. Agronomic Characteristics and Agricultural Concerns

**Figure 1:**
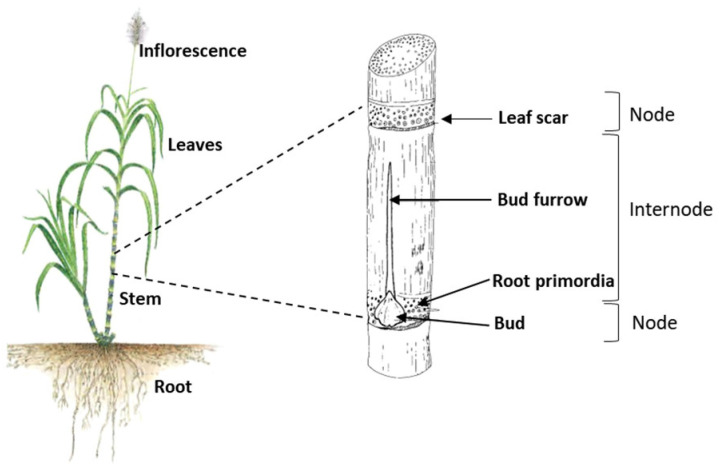
Representation of a sugarcane plant and a piece of cane stalk [13,24].

Modern sugarcane is a polyploid hybrid of species such as *S. officinarum*, *S. spontaneum*, *S. robustum*, *S. barberi* and *S. sinensi* [17,19]. This crop is one of the most genetically complex species due to its large chromosome numbers (Table 2). This perennial C4 plant produces efficient photosynthesis, especially at high temperatures, [23]. The stems grow in clumps, and each unbranched stem is a succession of nodes and internodes. The diameter of the stalk is about 2.5–5 cm and the length is 2–5 m. The leaves reach a length of about 30–100 cm and are alternatively attached to the stem at the base of the nodes (see leaf scar of Figure 1). The root system is composed of adventitious roots emerging from the stem and permanent fasciculated roots. The inflorescence, also called the arrow, is a ramified panicle of 25–30 cm in length.

Sugarcane cultivation starts with planting a stalk piece in the soil. Several stems grow to form a clump that ripens after 12 to 18 months [20]. The cane is mature, and therefore ready to harvest, when the sugar content at the top is almost as high as at the bottom. The plant–cane cycle ends after the first harvest, and the roots and lower parts of the plant are left in the field to allow the growth of new systems. After four or five ratoon–cane cycles, the crop is eradicated from the field by ploughing [11,16]. 

To fight pests (borer, nematodes, termites or beetles) and diseases, the breeding programs produced new resistant cultivars [23,25]. The other threats to sugarcane cultivation are weeds. For example, in Bangladesh, weed competition leads to yield losses of 40%, compared to 20% by pests [26]. The crop suffers from competition for water and nutrients from weeds, which provide a breeding ground for disease-causing insects.

To control invasive plants, the application of phytopharmaceutical products is widely favored. However, those practices depend on the regulations of the country. Over the last decade, in the EU, the list of approved herbicides, such as Atrazine, Asulam-based herbicides and glyphosate-based products, has decreased, triggering some difficulties in weed control [27,28,29]. The current general trend is to reduce the use of herbicides by adopting new weed control strategies, including innovative cultural and biological technology [25,30]. 

Pre-harvest burning of sugarcane is the most widely used weed control method, but it has a negative effect on SCJS’s quality. The study of Galinaro et al. indicates that the concentration of total polycyclic aromatic hydrocarbons (PAHs), restricted in food in the EU because of their toxicity, is more than 10 times higher in cachaça produced from burned sugarcane crops (21.1 μg/L) than from unburned crops (1.9 μg/L) [31]. Another study from Thai and Doherty reports that cane juice from unburned cane has a higher proportion of soluble inorganic ions, organic acids, proteins and polysaccharides than that from burned cane [32]. The concentrations of furfural and 5-hydroxymethylfurfural (5-HMF) are also higher in cachaça from burned sugarcane [8]. By contrast, manual weed control, mostly used by smaller producers, is effective, but it can be difficult for farmers to find the necessary workforce for this laborious task [11].

To summarize, weed management requires more diverse and effective methods to respond to the needs of all types of farmers [33]. Moreover, the other greatest current threats to sugarcane cultivation are related to climate change [34]. The lack of water and the increase in temperatures could negatively impact the sugarcane quality [30] and therefore threaten the production of SCJSs. For example, weeds such as *Rottboellia cochinchinensis*, *Ipomea plebeia* and *Digitaria sanguinalis* are expected to increase under high-temperature conditions [34].

### 2.2. General Uses and Chemical Composition of Sugarcane

**Figure 2:**
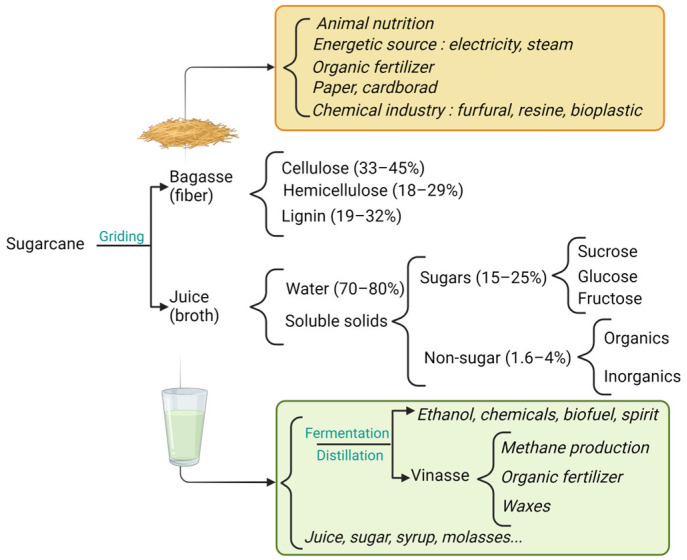
Composition and general uses (non-exhaustive list) of sugarcane. Created with BioRender.com. https://app.biorender.com/ (accessed on 23 June 2023).

Sugarcane is widely used industrially and both fractions obtained from the milling, juice and bagasse, have a wide range of applications (Figure 2). Cane juice is a nutritious energy drink, extracted simply by chewing [35] or with a mechanical crusher [36]. It represents 80–90% of the cane and it is composed of 70–80% of water, 15–25% of soluble solids composed of fermentable carbohydrates, 0.8 to 2% of other organic compounds (wax, amino acids and starch) and 0.8 to 2% of inorganics compounds (Figure 2) [37]. The fibrous residue (10–20%) called bagasse and containing at least 49% moisture, is composed of approximately 33–45% of cellulose, 18–29% of hemicellulose and 19–32% of lignin [31]. 

Sugarcane provides 60–70% of the sugar produced in the world and other various valuable products like jaggery, syrup, cane honey and molasses [35]. It is important to acknowledge the specificity of small territories such as islands where there is strong competition between producers using the same raw material. This leads to a reduction in the availability of sugarcane for SCJS’s production.

Sugarcane juice has a color ranging from light gray to dark green due to plant pigments (anthocyanins and chlorophyll), polyphenolic compounds, the degradation products of sugars condensed with amino derivatives, and the reaction of ferric salts with the tannins. Colloidal matter found in the fresh juice is responsible for turbidity [36]. 

The main compound is sucrose [37]. Its concentration increases from the stem bottom to the top during the growth. Starch is found in the stalk and in green leaves but is absent in roots. Its content varies during the day and it is converted into sugar during the night [19]. In the case of alcoholic beverage production, unlike sugar production, the non-sugar composition (vitamins, nitrogenous compounds, etc.) must be suitable for alcoholic fermentation, and this will impact the fermentation yield [20] (Table 3). 

As reported by Chen et al., the main aroma characteristics of sugarcane are green and grass odor [39] as contributed by 1-hexanol, *trans*-2-octenal, nonanal and *trans*-2-nonenal. Floral and honey aromas are associated with phenylethyl alcohol and 2-ethyl-1-hexanol. The content of carboxylic acids, alcohols and esters increases in the juice, and some compounds not detected in sugarcane are present such as caprylic alcohol, d-limonene, ethyl palmitate and *trans*-2-nonenal. This latter compound could appear due to the microbial and enzymatic effects during the juice extraction (Table 4).

## 3. Styles and Methods of Production of SCJS

### 3.1. The Notion of Terroir

Terroir is a French word that has the Latin root Terra meaning ‘earth’ or ‘soil’ [40]. Frequently used in the wine industry, the notion of terroir refers to the natural specification linked to the geographical origin of the product [41]. This concept makes it possible to define characteristics that influence or explain the unique sensory and chemical profiles of a wine and has been also revealed for several spirits such as whiskey [42], cognac [43] or cachaça [44]. Producers of agricultural rum from Martinique have made use of this concept to differentiate their products thanks to the AOC designation [5].

**Figure 3:**
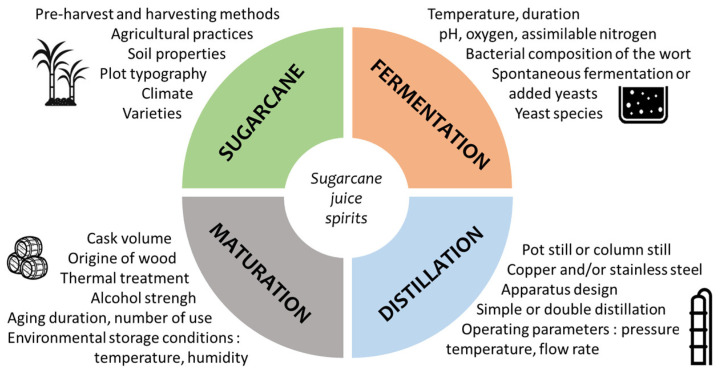
Factors influencing the chemical and sensorial profile of SCJSs.

Hence, the diversity of the SCJS sensory profile depends not only on the terroir-related elements but also on each step of the production process (Figure 3). For a freshly distilled spirit (non-aged SCJS), the factors connected to the sugarcane can greatly influence the flavor [31]. Furthermore, fermentation [45,46,47,48] and distillation [49,50,51] parameters play an important role in the organoleptic characteristics of the spirit. For aged SCJSs, the different conditions of maturation in wooden barrels define the quality of the product [10,52,53,54,55]. 

### 3.2. Sugarcane Juice Spirits in the World

**Figure 4:**
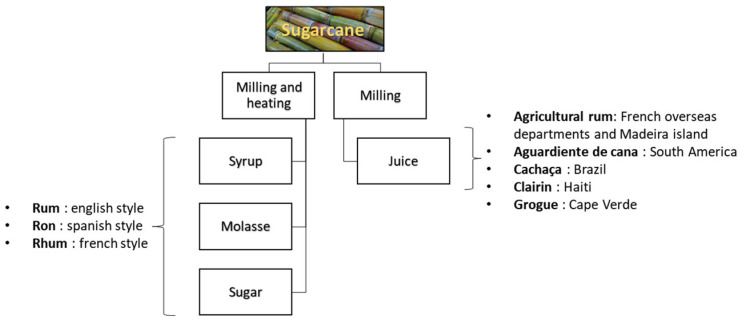
The different spirits made from sugarcane.

The large family of sugarcane spirits includes those made from the thermal processing of the juice (Figure 4). The area of production determines the designation of ‘rhum’, ‘ron’ or rum according to whether the production style is French, Spanish or English [56,57]. Regarding the other category of sugarcane spirit, those made from the pure juice are also diverse with their own denominations and specific organoleptic properties. 

**Figure 5:**
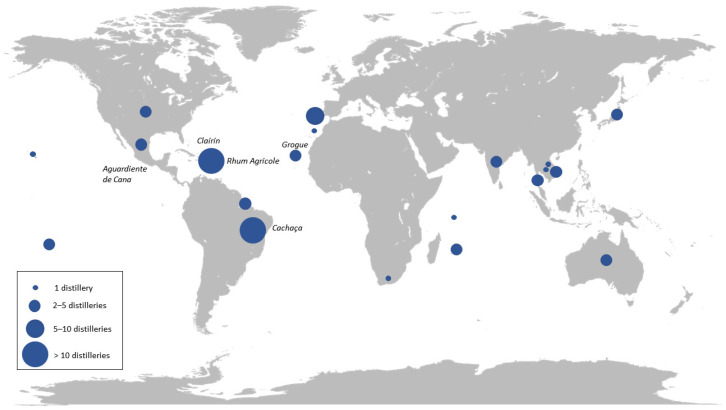
Production of spirits made exclusively with pure sugarcane juice over the world. Data were collected in February and July 2023.

Nowadays, SCJSs are produced worldwide. Figure 5 presents only those produced with pure sugarcane juice (non-heated). This list was compiled by searching spirits vendor websites, producer websites and books. This narrows the list considerably, as some producers blend SCJSs with molasses. The most famous SCJSs are cachaça, a Brazilian spirit and rum produced mainly in the Caribbean with hundreds of artisanal or industrial producers. Clairin, from Haiti, is produced with hundreds of units. On the other side of the Atlantic Ocean, in Cape Verde, the grogue with only pure cane juice is produced by two distilleries. We can see that the emergence of new SCJS producers in areas dominated by molasses-based rums is slowly changing the market. However, for the moment, in the Asia/Oceania area, 13 distilleries producing pure cane juice alcohol are reported with 3 distilleries in Japan and Vietnam; 2 distilleries in Thailand, the 5th largest producer of sugarcane in the world, as well as Australia; and 1 distillery each in India, Cambodia and Laos. In the Indian Ocean, the islands of Mauritius (three), Reunion (three) and Seychelles (one) accounted for seven distilleries. Only one distillery producing pure cane juice rum could be identified in South Africa. On the American continent, there are, respectively, three and two distilleries located in the USA and in Mexico. And finally, in the Pacific Ocean, four distilleries in Hawaii (one) and Tahiti (three) produce SCJS.

The following chapters briefly present two well-documented SCJSs, Rhum Agricole and cachaça, and three other SCJSs, which are less documented in the literature: clairin, grogue and ‘aguardiente de cana’.

### 3.3. Rum and Rhum Agricole

According to European law, rum is exclusively produced by the distillation of fermented material issues of sugarcane (*Saccharum officinarum* spp.) such as the molasses, the syrup, or the juice. It is distilled at less than 96% ABV and has the specific organoleptic characteristics of rum. The rum is not flavored and the alcoholic strength is at least 37.5% ABV [58]. The term ‘traditional’ could be added for an unsweetened rum obtained by distillation at less than 90% ABV with a non-ethanol volatile substance content minimum of 225 grams per hectoliter of pure alcohol (HPA). The definition of rum by the CARICOM standard (Caribbean Community) is very closely related to the EU standard [59]. Even for the definition from the US, rum is a spirit distilled from fermented sugarcane material such as juice, syrup, molasses or other by-products or the cane sugar itself [60].

The history of rum started with the huge trade based on cane cultivation and the sugar industry in the 16th century [60]. The ancestor of rum, made from molasses, bears different names over time and location: ‘Rumbullion’, ‘Rumbustion’ and then ‘kill-devil’ in the English-speaking regions [60,61]. In the Spanish-speaking regions, the spirit was called ‘aguardiente’ or ‘guaro’. The term ‘Guildive’ or ‘Tafia’ was used in the French-speaking areas [62]. The predominant raw materials used to produce this spirit were the molasses and syrups imported from the neighboring Caribbean islands and the USA [63]. Halfway through the 19th century, a new method of rum production was developed. Some farmers isolated from the production units choose to use their own cane to produce rum from the juice instead of the molasses. This is the ‘z’habitant’ rum (creole word), the ancestor of Rhum Agricole [62]. The term ‘Agricole’, which means ‘agricultural’, can only be used as a geographical indication for traditional rum produced from the sugarcane juice of French overseas departments (Guadeloupe, Martinique, French Guyana and Reunion) or the autonomous region of Madeira [10]. Agricultural rum is characterized by sugarcane fresh notes, not found in rum made from molasses or syrup [57]. Martinican distillers protect their spirit with the AOC label. The decree, which was enacted on 5 November 1996, describes the conditions to be respected, presented in Table 5, so that Rhum Agricole can obtain the AOC Martinique label. This spirit is produced with pure and fresh sugarcane juice without the addition of syrup and molasses. The aged rum is obtained exclusively in oak barrels and the addition of caramel is prohibited.

This alcoholic beverage is economically important for French overseas departments such as Martinique and Guadeloupe, which produced, respectively, 100,000 and 80,000 hectoliters of pure alcohol (HPA) in 2021. French rum exportation is flourishing with an increase of 24 million euros between 2020 and 2022 [65]. This positive outcome has not been the case for rum producers in other parts of the world after the two years of pandemic. Other producers worldwide have registered a decrease in sales of 70% in 2020 [66].

### 3.4. Cachaça

The white cachaça (non-aged), called also ‘pinga’, is produced by about 40,000 Brazilian distilleries, but only 955 with a valid registration [8] This spirit is produced either by an industrial method (75%) with modern scientific techniques using stainless columns or by an artisanal method (25%) with ancestral techniques with a copper alembic [6]. 

According to Brazilian legislation, the term cachaça is a sugarcane spirit containing 38–48% ethanol (*v*/*v*) at 20 °C obtained by distillation, with a column or pot still, of fresh fermented sugarcane juice and produced only in Brazil [6]. The aged cachaça is matured in wooden barrels (maximum capacity of 700 L) for at least one year [67]. Oak as well as tropical local woods such as amburana, bálsamo, jatobá or jequitibá are used for maturation [68,69,70]. This very popular traditional drink in Brazil has recently begun to be exported internationally and Brazilian producers are seeking to increase the export rate, rising from 1% of exportation 20 years ago [71] to 50% today [72]. Over the past 20 years, Brazilian scientific researchers have been acquiring data to provide producers with valuable new tools to optimize their production methods [7]. Brazilian legislation defines the rules to produce cachaça (Table 6) and MAPA (‘Ministério da Agricultura Pecuária e Abastecimento’) suggests the use of analytical techniques suitable for sugarcane spirits for quality control.

Twenty years ago the annual production of cachaça was estimated at 1.3 billion liters per year [73], and nowadays it reaches about 2 billion liters, representing an economic activity estimated to be USD 6 billion [74].

### 3.5. Other SCJS

Unlike other spirits, such as whiskey or cognac, sugarcane spirit factories often lack technical knowledge; production can be empirical and rudimentary. Nevertheless, the situation is changing with the development of spiritourism as an economic actor for developing countries. For the following spirits, which are less commonly described in the literature, some producers are seeking to maintain their traditional production methods while striving to ensure superior product quality.

Aguardiente de cana refers to those based on sugarcane, whether juice or by-products, in Spanish-speaking regions. Aguardiente, meaning firewater, is produced in artisanal distilleries in Central and South America with only sugarcane juice [75,76]. These pure cane juice rums are less found on the international market. 

Clairin is a traditional rum made according to traditional methods in Haiti by hundreds of rural distilleries. The spontaneous fermentation is realized by indigenous yeasts in wooden vats and lasts several days with fresh cane juice or cane syrup [77]. Moreover, aromatic plants or fruits can be added. The distillation is performed with a pot still called Charentais copper alembic. The first distillate is between 25 and 35% ABV and can undergo a second distillation to reach a higher alcohol content [20].

Grogue called also, ‘grogu’ or ‘grog’ is a traditional rum from Cape Verde. On the archipelago, the island of Santo Antão produces a large quantity of sugarcane on field terraces. Cutting and cane juice extraction is often manual. The fermentation is achieved in days (8 to 15) to weeks. The distillate with a 38–54% ABV is usually obtained using a copper pot still and is mostly consumed locally unaged. However, after a maturation period of 2 to 3 months, the grogue can be aged in wooden barrels [78].

The lack of legal requirements regarding the spirit-making practices in microdistilleries can lead to the manufacture of poor-quality products [79] that can even be harmful to health (high acetone or methanol content). The study of Pereira [80], based on Brazilian legislation, shows the difference in chemical profile between grogue and cachaça from artisanal and industrial production. Thus, copper levels above the Brazilian limit of 5 mg/L were reported in several artisanal production units. Moreover, the chemometric analysis (PCA analysis) performed on both spirits in 2012 shows a greater dispersion of the chemical profiles of grogue, probably due to the lack of regulation at that time [81]. Since then, Cape Verde began to regulate the production of this traditional spirit. The legislation of 12 August 2015, and the national program Vagrog II, is aimed at obtaining standardization and promoting grogue on the international market [82].

### 3.6. Traditional Customs and Uses

Because of their high alcohol content, distillates were first used medicinally [1]. Medicinal plants are used in maceration with spirit as an excellent hydroalcoholic solution for the extraction of natural compounds [83]. For example, the Bay-rum, an anti-rheumatism lotion, used for local application in the Caribbean, is obtained by maceration of West Indian bay-tree (*Pimenta racemose*) leaves soaked in rum for several weeks under the sun [84]. In Haiti, clairin is used to make ‘Trempé’, a maceration of wood extracts and spices in 22% alcohol strength clairin [85] and in Voodoo rituals [86]. Another more widely known example is the grog made of rum and hot water with lemon and cinnamon as a flu treatment.

Over the past decade, there has been a renewed interest in traditional medicines. To guarantee the safe use of these homemade medicines, studies have been carried out on the therapeutic and toxic properties of plants and their extracts in hydroalcoholic solutions such as rum [87]. Maceration of aromatic plants in spirit can provide the beverage with several positive characteristics when it is consumed in small quantities thanks to plant phenolic compounds as a source of antioxidants [88] and extracted volatile and biologically active compounds. 

SCJSs as a regional cultural heritage are widely used in gastronomy, to flavor cakes and pastries, and are consumed neat, on ice and in traditional cocktails. The ‘Caipirinha’, made with cachaça, sugar, lime juice and crushed ice [89], the ‘Ti’Punch’ (Rhum Agricole, sugar and lime zest) from Martinique, or the ‘Punch’ (Grogue, honey, lemon and cinnamon) from Cape Verde [63] are emblematic beverages representing the country’s image. In accordance with the concept of food pairing, marketers approach new consumers by introducing them to new flavors.

### 3.7. The Sugarcane Juice Spirit Process

The quality and sensory profile of SCJSs depends upon the different steps of the process. The type of harvest (manual or mechanical), the pre- and post-treatments of the cane during the milling, the alcoholic fermentation conditions, and the type of distillation apparatus will define the chemical profile of non-aged SCJSs. Finally, the maturation in wood barrels is the last step that transforms, improves and enhances the sugarcane distillate. For the different SCJSs cited here, the diversity of flavor can be explained by the specificities of the making process presented in Table 7.

#### 3.7.1. From the Field to the Mill

Once mature, sugarcane stalks are harvested manually with a cutting instrument called a machete or mechanically with a cane harvester. Burning cane pre-harvest facilitates manual cutting by reducing worker injuries from sharp leaves, insects, and snakes, but this agricultural practice has an impact on the atmospheric environment [90] and on the juice’s chemical composition. The burning of sugarcane is seldom practiced today because it reduces the quality of the stalks and destroys the biological balance in the fields [90]. During the harvest, cane top and leaves are separated from the stalk because they decrease the sucrose yield [19]. The mechanization of sugarcane harvest has advantages, reducing the effort of manual harvest and increasing the yield of homogeneous stalks for easier grinding. However, some disadvantages must be considered: a higher deterioration in cut cane and lower quality caused by non-cane materials not deliberately harvested with the cane (leaves, invasive plants and mud) [91]. The whole cane stalks (manual cutting) or in sections (mechanical cutting) are then rapidly transported to the distillery to reduce the impact of post-harvest sugar loss. 

The juice extraction efficiency has greatly increased since the very first two-roller mill was created in India in the 1500s [92]. During the 17th century, the Brazilians improved their mills to increase grinding efficiency and to reduce the loss of the slave workforce. In the East, the first mills with two vertical gears, developed by the Chinese, were introduced in Japan in 1610. The origin of the three vertical gear mills is believed to be in Peru and in Mexico back in the 1700s. Nowadays, the animal-driven models with vertical gears can still be found among the small-scale producers, whereas the mills with three horizontal gears are still mainly used in modern distilleries. Motor-driven extraction can be improved using a cane kicker, composed of sets of knives, which breaks up the stalk and promotes regular feed on the conveyor. Then, a shredder, composed of a feed drum and a rotor with hammers, achieves the cellular rupture [93].

The addition of water, called imbibition water, during grinding, improves the efficiency of sugar extraction reaching 92 to 96% for the modern mills [94]. This dilution allows the control of the sugar content and, therefore, defines the alcohol content in the cane wine. Limiting the quantity of sugar available in the fermentation medium helps avoid reaching an alcohol concentration that inhibits yeast growth [95]. Particular attention must be paid to its chemical and microbiological composition because this water is part of the composition of the wort and will therefore have an impact on yeast fermentation efficiency. It is recommended not to use water with a high mineral content that may contain a higher bacterial charge [96]. Composed imbibition refers to the return of the juice from the last mill to the first mill. This technique has the advantage of improving the total extraction of juice [97]. The hygiene of the crushing equipment also has an impact because the microorganisms found on the surface of the rolls will be part of the wort microflora. 

Motorized extraction requires a significant input of energy. According to Inskip, the energy requirement is estimated between 28.6 and 43.7 MJ for a milling rate of 400 t/h and a 15% fiber cane [93]. 

To eliminate the fine bagasse particles found in the juice, post-grinding filtration or decantation techniques such as a strainer or rotary vacuum-drum filter can be used. Then, to perform the fermentation, the sugarcane juice can be supplemented with several components and is, thus, called wort or must. Sulfuric acid or citric acid can be added to adjust the pH and thus regulate the aerobic bacterial flora [20]. With the same aim, disinfectants or antibiotics such as hydrofluoric acid, sodium fluoride and sodium penicillin G can be added [20]. In the case of grogue, the addition of sugar of less than 6 g/L is authorized in the wort [78].

#### 3.7.2. The Key Step of Fermentation

This is the step where secondary products, responsible for sensorial characteristics of SCJSs, are formed in addition to the alcohol. Yeasts, generally belonging to the genus *Saccharomyces* [98], perform the biochemical reaction of transforming carbohydrates (sugars) into ethanol and carbon dioxide to produce energy released as heat. Sucrose, the main sugar in sugarcane, cannot be catabolized directly by the yeast, so it is first converted into glucose and fructose (by consuming ATP). Once activated in phosphate form, glucose and fructose enter the glycolysis pathway cycle (Embden–Meyerhof) to give pyruvic acid [20]. Under anaerobic conditions, this pyruvic acid is reduced to lactic acid or ethanol after decarboxylation. In addition to the ethanol produced, by-products are released into the fermented juice, including acids, which leads to the acidification of the medium [98]. During a 24-h fermentation, most of the sugars are consumed in the first 12 h [99]. However, the fermentation can last days or weeks, especially with wild yeasts [20]. Vats used for fermentation are usually open and the site is ventilated to facilitate the removal of carbon dioxide. The most common vat material used in modern units is stainless steel, but wood or mild steel are also used [100].

In the case of spontaneous fermentation, no selected yeast strains are added. The heterogenous microbiota (yeasts and bacteria) from the cane, as well as those from the equipment, represent the ecosystem liable for flavor formation [46]. Thus, contamination by tools such as the mill, should also be taken into consideration. The surface bacterial microflora of the stalks, which can be found in the wort, is composed of aerobic lactic acid bacteria (*Corynebacteria*, *Micrococcus*, *Enterobacteria*, *Bacillus*, and *Pseudomonas*) and microaerophilic bacteria (*Lactobacillus*, *Leuconostoc*) [20]. These microorganisms have access to the sugarcane during stalk cutting and after insect attack. They proliferate if the cut cane is not crushed quickly [96]. The quality and quantity of bacteria in the wort flora depend on the sanitary status of the cane and the wort components. In the case of spontaneous fermentation, the conditions must be favorable to the dominance of yeasts in the wort. Among the bacteria encountered in cane wort, three families are often responsible for a decrease in spirit quality. Lactic acid bacteria (LAB) compete with yeast for sugar and nutrients [45,99]. An excessive proliferation of these bacteria will negatively influence the quality of the spirit because of the important production of 2,3-butandione, acids (lactic, acetic and formic) and acetoin. Bacillus, which forms a pellicle on the vat surface during long fermentation, adversely affects the yield by consuming ethanol and releasing higher alcohol and fatty acids [11]. At the end of the fermentation, when the carbon dioxide is at a low level in the wort, acetic acid bacteria can develop [45].

Nowadays, to ensure better reproducibility and control of fermentation, yeasts are added in different possible forms: mixed or pure culture, in dehydrated form, or as liquid ferment. In general, producers select yeasts with good fermentation yields, low hydrogen sulfide (H_2_S) production, killer activity (toxin production that eliminates sensitive strains), and the ability to flocculate and produce a high level of aromatic compounds [45]. The main yeast species used is *Saccharomyces cerevisiae*. Back in the 1970s, in the French West Indies, dehydrated baker’s yeast was used to allow better control of fermentation at a reduced cost. Another way to inoculate the wort consists of selecting and isolating endogenous strains that are adapted to production conditions [95,101], providing an inoculum also related to the terroir. Whatever the inoculation method, in the case of continuous fermentation, the sedimented inoculum can be recycled for other fermentative processes [102]. On the other hand, when fermentation is discontinuous (batch fermentation), the wort must be inoculated at each new vat filling. This method is less economical and substrate inhibition can occur with a high sugar concentration. Although the yeast species mainly used is *S. cerevisiae*, it is also possible to use non-*Saccharomyces* yeast. This could provide a different sensory profile. Amorim et al. evaluated that the addition of *Meyerozyma caribbica*, known as *Pichia* [103], influences positively the chemical and sensory quality of cachaça [58]. 

Besides the microflora impact, fermentation efficiency also depends on many physicochemical parameters such as temperature, pH, oxygen and nutrient availability to maintain the growth of yeasts and their viable population, until the consumption of all sugars contained in the wort (Table 8). The nitrogen content available for yeast metabolism in cane juice depends on the cane cultivar and agronomic conditions [104]. When free amino acids are not sufficient for optimal yeast growth, nutrients such as ammonium sulfate can be added to the wort [102]. However, excessive nitrogen content and, conversely, very low nitrogen concentration can induce a high production of higher alcohols [104]. For cachaça, a fermented mixture of crushed corn, lemon and rice is added as a source of supplementary substrates [101], and yeast extract as a source of nitrogen [102]. 

High temperature due to exothermic reactions during fermentation promotes yeast autolysis, the growth of thermophilic bacteria such as lactic and acetic bacteria [20], and decreases the survival rate for yeast in an environment containing a high ethanol concentration when the temperature is higher than 35 °C [99]. To control the fermentation temperature, moderate-volume vats must be installed in a cool and airy space. The use of a cooling system is also possible. A low temperature can induce sluggish fermentation because of a decrease in membrane fluidity and temperature-dependent enzyme activity as permease [105]. Temperature control is even more important when fermentation is spontaneous to avoid the production of undesirable compounds such as acrolein [8]. 

Oxygen must be provided at the beginning of the fermentation cycle or before the pitching of the wort (preoxygenation) [106] to promote the biosynthesis of unsaturated fatty acids and sterols responsible for the fluidity and permeability of the cell membrane. Yeasts are then better protected against oxidative stress. However, an excess of oxygen can cause the formation of reactive oxygen species (ROS) that will cause oxidative damage to the cell [106]. 

The juice extraction and fermentation stages are considered to be those responsible for the highest losses in alcohol yield (between 25 and 50%) [20,96]. A better understanding and control of factors influencing fermentation would improve alcohol yield and spirit quality. 

#### 3.7.3. The Art of Distillation

Distillation is a physical process that can separate a mixture of liquids based on the differences in boiling point and volatility of compounds [107]. The process involves heating the liquid mixture, the cane wine, and then condensing the vapor. These two successive steps, evaporation and condensation, can be performed as a continuous or discontinuous process using a column still or a pot still (alembics) [8]. Throughout the industrialization era (18–19th centuries), many major improvements were performed in the Caribbean and South America, increasing the production and quality of sugarcane spirit [1,95]. From the 20th century onwards, both continuous and discontinuous distillations were perfected. 

Depending on the still and its characteristics (copper or stainless steel material, size, design and distillation parameters), the spirit will have a different sensory profile [95].

**Figure 6:**
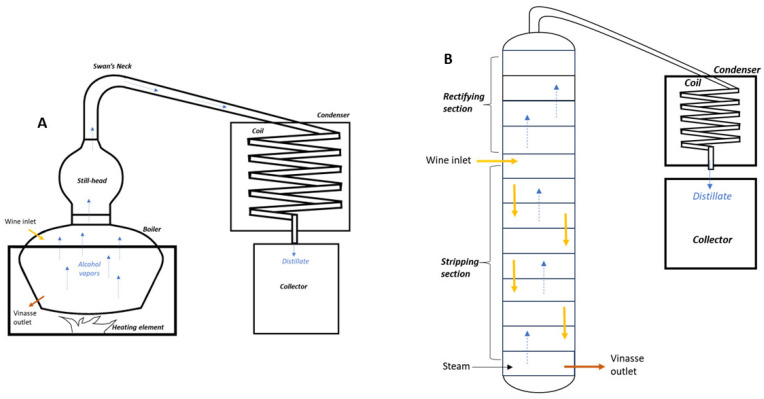
Simplified scheme of copper still (**A**) and column still (**B**).

The discontinuous distillation in a batch or pot still starts by charging the pot with wine and heating it. Alcohol vapors, richer in volatile compounds, reach the top of the pot (Figure 6). The reflux stream returns to the pot and the distillate is collected in a storage tank. Three fractions or cuts are collected in separative receivers depending on the boiling point of substances [6]: the head, the heart, and the tail [108]. The heart fraction corresponds to 80–85% of the total distillate volume [95]. Only the heart fraction, which contains the more suitable volatile compounds, is kept. The head fraction (1 to 5%), containing mostly methanol, and the tail fraction (15%), composed of higher alcohols such as propanol, butanol, and isobutanol (fusel oil) and aldehydes, are discarded [95]. The double distillation allows lighter aroma spirit with a higher alcohol content to be obtained [108]. It is also used to reduce the concentration of ethyl carbamate above the limit of 210 μg/L (Table 6). In this case, the first distillate is not split and the three fractions are separated during the second distillation [6].

The column still contains several trays, and the wine is introduced continuously to the column. The column is divided into two sections: the rectifying section, above the entry of the wine inlet, and the stripping section, below (Figure 6). Steam injected on the bottom tray reaches the top of the column through the bubble-cap trays. The vapor at the top of the column, richer in alcohol and other volatile components, is condensed in the condenser [8]. A part of the condensed vapor, called reflux (rectification), returns to the column [1], and a degassing system can be added at the top of the column. This method of distillation, favored by medium and large-size distilleries, has the advantages of being energy-saving and improving the standardization of distillate [95].

During the distillation process, in addition to the separation of compounds according to their boiling point and their concentration, other reactions contribute to the development of unique organoleptic properties. Esterification reactions between alcohol and acids, Maillard reactions, and oxidation reactions of sulfur compounds participate in the formation of aromas [20]. Moreover, copper contributes to superior product quality by forming insoluble copper salt and neutralizing the unpleasant flavor of sulfur compounds [7,8].

The distillate is stored in a tank, whereas the vinasse is discarded or treated to limit its impact on the environment [109]. In the case of efficient distillation, the output vinasse should contain a low ethanol content of less than 0.6% [110]. The fresh distillate has a harsh flavor that can be reduced after a few weeks of resting in an inert container (stainless steel) [6]. Stirring could help eliminate some small off-flavor volatile compounds [20].

#### 3.7.4. Aging

The physicochemical profile (color and flavor) is modified during aging due to the biochemical reactions between the spirit and the wood [67]. The wood widely used to age SCJS is oak (*Quercus* spp.). However, for cachaça, in particular, many local woods are used, thus bringing aromatic profile diversity [53,111]. The different compounds of wood, cellulose, hemicellulose and lignin interact with the alcohol and generate new compounds [112]. Depending on the duration and intensity of toasting, the spirit’s aromatic profiles will be different [53,112]. The ‘char’ treatment corresponds to an intense but short toasting of the wood [113], whereas the ‘toast’ treatment is less drastic with several levels of intensity. 

The first elements that appear after filling the barrel are color and tannins [114]. During the aging process, different biochemical reactions occur depending on three main parameters [67,115]: the barrel features, the duration and conditions of storage [112] and the initial composition of distillate [116]. 

Aged spirit profile varies according to the wood species (permeability, porosity and density), size and geometry, thermal treatment and number of uses of the barrel [117]. Depending on the grain of the wood, more or less tannins will be released [118]. The coarser and wider the grain, the more tannins will be found in the alcohol. The porosity of the wood allows the interaction of oxygen with spirit compounds. The resulting oxidation removes the harshness of the alcohol, increases the fruitiness (esterification) and enhances the complexity. The cellulose is not normally degraded by heat treatment during barrel making [52]. Thermal degradation of hemicellulose and lignin generates new compounds as presented in Table 9. The evaporation rate of the liquid will be influenced by the size of the barrel, the temperature, the humidity level, the barrel pre-treatment and the air circulation in the cellar.

The climate (temperature, humidity and atmospheric pressure), sometimes difficult to control, plays an important role in maturation, especially in the evolution of the alcohol content in the barrel. The alcohol concentration decreases during the aging process because of its passage through the pores of the barrel and the reactions of ethanol with secondary compounds. Douglas Santiago et al. indicate that during an aging process in high humidity conditions, the quantity of alcohol decreases, whereas when the humidity is low, it is rather the water that evaporates and the alcohol content of the spirit is higher at the end of storage in the barrel [119]. This study states that 3 to 4% of alcohol is lost annually in the barrels in Brazil. In tropical areas, this evaporation rate, called the angel’s share, can reach up to 10% in cellars under uncontrolled temperatures where the temperature ranges from 25 to 35 °C with a rather high relative humidity between 65 to 90% [117,119]. Moreover, in these favorable conditions to alcohol evaporation, the biochemical reactions are faster. Thus, the extraction of phenolic compounds is accelerated and spirits mature 3 to 4 times faster than in temperate climatic zones [67]. Therefore, it is important to study the kinetics of maturation-related congener formation under real production conditions [10]. According to the daily and seasonal temperature changes, the liquid contained in the barrel will undergo expansions and contractions, which will participate in the elaboration of the flavors [119]. The study of underwater aging of bottled agricultural rum by Aguiar et al. reports the positive impact on sensory characteristics without the participation of compounds from wood [10]. 

During the aging process, three types of mechanisms occur: subtractive, additive and interactive (Table 9). Subtractive mechanisms correspond to the capacity of the carbonized wood to purify the spirit. The carbon removes immature characteristics from the distillate, such as off-flavor sulfur derivatives (e.g., H_2_S, dimethyl disulfide) [120]. Additive mechanisms allow the modifications of flavor, texture and color of the spirit by extraction of volatile compounds, phenols, sugars, glycerol, non-volatile organic acids and tannins from the wood. Interactive mechanisms refer to polymerization, esterification, hydrolysis and oxidation reactions. The wood improves the organoleptic characteristics of the spirit by reducing its aggressive and bitter character.

The cellar master can blend different batches to obtain the desired sensory profile using, for example, the solera method, which corresponds to blending different ages. In the cellar, the casks are classified into levels based on the age of the spirit. The first level (Criadera) receives the fresh distillate and the last levels contain the oldest spirit (Solera) [56,121].

**Table 9:** Biochemical phenomena occurring during the aging. A: additive; I: interactive and S: substractive mechanisms.

Compound	Evolution	Biochemical Reaction Involved	Ref.	A	I	S
**Sulfuric compounds**	↓	Interact with copper—High volatility	[122]			X
**Copper**	↓	Absorbed or adsorbed by the wood. Reaction with phenolic compounds	[67]		X	X
**Ethanol**	↓	Evaporation, oxidation, reaction with secondary compounds	[67]		X	X
**Aldehyde (Ethanal)**	↑	Oxidation of amino acids, alcohols, and fatty acids	[67]		X	
**Esters (Ethyl acetate)**	↑	Esterification of fatty acids with ethanol	[67]		X	
**Acetic acid**	↑	Due to ethanol oxidation—Degradation of hemicellulose	[67]	X	X	
**Volatile acidity**	↑	Acetic acidity increasing and transfer of non-volatile organic acids from the barrel to the beverage	[67]	X	X	
**Methanol**	↑	Degradation of hemicellulose (pectin)	[67]	X	X	
**Ethyl carbamate**	↑	Conversion of cyanide to cyanate by the action of peroxides	[67]		X	
**Tannin**	↑	Extraction from the wood	[123]	X		
**Gallic acid**	↑	Hydrolysis of wood tannins → increase viscosity	[67]	X		
**Furfural and 5-HMF**	↑	Thermal breakdown of pentoses and hexoses from hemicellulose	[67]	X		
**Cinammic aldehydes**	↑	Coniferaldehyde and sinapaldehyde generated from lignin	[10]	X		
**Vanillin, syringaldehyde**	↑	Derived from lignin; oxidation of sinapaldehyde and coniferaldehyde	[10]		X	
**Vanillic acid, syringic acid**	↑	Derived from lignin; oxidation of vanillin and syringaldehyde	[123]		X	
**Phenolic compounds**	↑	Simple extraction (low molecular weight) and extraction and hydroalcoholic degradation of lignin	[123]	X	X	
**Sugars (glucose, arabinose, sucrose, maltol, xylose, etc.)**	↑	Hydrolysis of tannins, extracted from the wood, hemicellulose degradation	[20,123]	X	X	
**Glycerol and fatty acids**	↑	Extraction and/or hydrolyzed of resins and triglycerides from cell walls of wood	[123]	X	X	
**Coumarin**	↑	Formed by the cyclization of o-coumaric acid	[123]		X	

#### 3.7.5. Quality Control during the SCJS Making Process

Most spirit producers obtained their expertise through many years of successful and failed experiments. Hence, the determinants of a spirit’s flavor can be random without standardization. For this reason, quality control must be carried out at every stage of spirit production to guarantee long-lasting quality and yield. However, depending on the technical, human and financial resources, small to large-size producers do not have the same capacities to ensure this control. Table 10 lists the analysis and associated techniques for monitoring the production from the field to the bottle. The method’s global cost includes the price of equipment, reagents and laboratory consumable items needed. The technical skill necessary is also estimated.

Sugar and nitrogen contents are good indicators for evaluating the cane’s ripening. During plant growth, the sugar concentration increases progressively from the bottom to the top while the amino acid concentration decreases. Evaluation of the sugar content of the juice can be performed quickly with a Brix refractometer. Brix value can also be used to monitor the fermentation process and determine the mill efficiency by analyzing the juices from the successive rolls. Bagasse humidity is an indicator of potential fuel value and mill extraction efficiency. The juice pH, slightly acidic due to organic acids, is measured as an indicator of freshness when the cane arrives in the distillery [5]. A significant decrease in pH can be a sign of the beginning of fermentation and, therefore, a warning of bacterial contamination.

The physicochemical conditions of fermentation can be followed by performing different analyses on both wort and wine. These elements will indicate if the fermentation is over (density, Brix, total reducing sugar and nitrogen) and detect bacterial contamination (acidity and pH) [99,102]. Total acidity (volatile and fixed) is a crucial parameter in control quality [8]. Thus, according to Brazilian legislation, the volatile acidity of a wine, expressed as acetic acid concentration, must be below 150 mg/100 mL aa (anhydrous alcohol). An evaluation of the health of the yeast population (adaptability, growth, etc.) can be defined by counting the number of cells, the rate of viability and their division rate (budding index). The assessment of fermentation efficiency and yield are determined by calculating sugars and alcohol contents in wort and wine using high-performance liquid chromatography with a refractive index detector (HPLC-RI) [48,124]. 

Distillation performance is determined according to three criteria: the chemical profile of the spirit, and the alcohol content in both the spirit and vinasse. It can be useful to define the composition of the organic and inorganic compounds of the vinasse, especially when it is intended to be applied in the cane field [109]. 

Several analytical techniques can be applied to distillate characterization. The determination of volatile organic compounds (higher alcohols, esters and aldehydes) is mostly performed by gas chromatography (GC) with a flame ionization detector (FID) or mass detector (MS), with or without derivatization [8,10,67,125,126,127], whereas the maturation-related congeners are analyzed by HPLC with ultraviolet (UV), diode array (DAD) or MS detectors [67,128,129]. These analyses estimate the aromatic richness and the presence of contaminants such as methanol and ethyl carbamate. A high ratio of total esters to ethyl acetate may be indicative of superior spirit quality [108]. Spectrophotometric analysis is useful to determine the colorimetric characteristics of the product and to follow the extraction of phenolic compounds (total phenol index). Not all analytical methods are equal in terms of precision and reliability. For example, the Folin–Ciocalteu method for phenolic compounds is cost-effective and requires little advanced equipment, but it has low specificity because reactions can occur with other reducing substances [123]. 

Inorganic compounds are determined by ion chromatography (IC), inductively coupled plasma (ICP) or atomic absorption spectroscopy (AAS) [51,80,130].

**Table 10:** Main methods of analysis used in control quality in a SCJS distillery.

Step of Process	Matrix to Be Analyzed	Analysis	Unit	Analytical Method/Apparatus	Cost	Technical Skills	References
Harvest/ Grinding	Cane juice	Brix	%	Refractometer—Brix spindle	+	+	[131,132]
		Sucrose	%	Polarimeter	+++	++	[131,132]
		Free amino acid	μL/mL	Spectrophotometer-UV	++	+++	[133]
		Purity	%	Calculation: P% = (Sucrose/Brix) × 100	+++	+++	[132]
Grinding	Crushed cane	Fiber	%	Weight and calculation	++	+	[132]
	Bagasse	Moisture	%	Moisture analyzer	++	+	[132]
Fermentation	Wort Wine	Density	-	Mustimeter/Densitometer	+	+	-
		pH	-	pH-meter	+	+	[134]
		Temperature	°C	Thermometer	+	+	-
		Total acidity	mg/L	Titration method	+	++	[134]
		Volatile acidity	mg of acetic acid/100 mL aa *	Titration method	++	++	[130]
		Budding index	%	Microscope	+	+	[99]
		Enumeration of yeast and bacteria	UFC/mL	Microbiological analysis	+	++	[99,100,101,102,103]
		Cell count	Cell/mL	Microscope/Neubauer chamber/Malassez cell	+	++	[102]
		Cell viability	%	Eritrosin/methylene blue	+	++	[102]
		Alcohol content	% (*v*/*v*)	Ebulliometer/Distiller	++	++	[61]
		Total nitrogen	mg/L	Kjeldahl method	++	++	[102]
		Sugar: glucose, fructose, sucrose	mg/L	HPLC-RI	+++	+++	[99]
		Total reducing sugar (TRS)	g/L	DNS method	++	++	[135]
Distillation	Vinasse	Alcohol loss	%	GC-FID	++	++	[110]
		DCO, DBO5, N, P, K	g/L or mg/L	Mostly spectrophotometry methods	+++	+++	[136]
Distillation Maturation Aging	Distillate (SCJS)	Inorganic components (anion, cation)	mg/L	IC/ICP OES/AAS	++	+++	[80,137,138]
		Ethanol content	% (*v*/*v*)	Densitometer/Hydrostatic balance/Pycnometer/spectrophotometric method	++ to +++	+ to +++	[73,102,131,139]
		Volatile acidity	mEq/L acetic acid	Titration method	++	++	[102]
		VOCs content	μg to mg/L	GC with FID/MS	++	+	[67,127]
		Ethyl carbamate	μg/L	GC MS/HPLC MS	+++	+++	[74,140]
		Matured-related congeners	μg/L to mg/L	HPLC—fluorescence/UV/DAD/MS detector	++	+	[52,67,127,130]
		Antioxidant activity	% inhibition	DPPH	++	+	[141,142]
		Antibacterial activity of phenolic extract	μg/ml	Bacteria culture. Minimal inhibitory concentration	++	++	[141]
		Dry extract	g/L	Drying under stirring	++	+	[116]
		Chromatic characteristics	-	CIELab method/Spectrophotometer	++	+++	[114,116,139,143]
		Total phenolic index	OD/g	Spectrophotometer at 280 nm	+	+	[114,116]
		Total phenolic compounds	mg/L	Folin–Ciocalteu method	++	++	[123]
		Dissolved oxygen	mg/L	Fluorometer	+++	++	[114]
Sensory Evaluation	SCJS	Odor activity	-	Odor thresholds—GC-olfactometry	++	+++	[71,144,145]
		Descriptive tests	-	Panel of experts	++	++	[74,122,146,147]
		Hedonic test	-	Consumers	+	+	[102,103,122]
		Difference test: triangle test	-	Consumers or experts	+	+	[71,148]

* aa: anhydrous alcohol; Estimated cost, +: EUR < 1000; ++: EUR 1000 to 5000; +++: EUR > 10,000, Technical skills required; +: very easy; ++: basic technical knowledge; +++: important technical knowledge.

It is essential to define the sensory profile of the spirit. Relating the chemical composition to the aroma profile is complex due to masking, additions and synergy phenomena. The sensory test is the easiest and fastest way to identify flaws [95]. Defining the visual characteristics is important because the golden color of aged spirits has an attractive impact on consumers. The flavor description is essential to guide them to choose a product according to their preferences.

Depending on the type of sensory test, it is necessary to define a common vocabulary to describe aromas [146] with an expert panel, to facilitate the descriptive analysis [149]. Aroma references can be prepared in a hydroalcoholic solution with an alcohol strength equivalent to the samples to be tasted (between 30 and 55% ABV) [150]. The orthonasal odor thresholds can be determined using reference standards (Table 11) [144,145,151]. The fresh-distilled SCJS is characterized by herbal, fruity (citrus, tropical fruit) and floral aromas. In aged SCJSs, the main aromas are spicy (clove, cinnamon and vanilla), empyreumatic (toasted, smoky, chocolate and coffee) and woody. Aromas perceived as flaws can give clues on the causes (bacteria contamination, incorrect distillation parameters) of poor sensory quality.

## 4. Chemical Composition and Their Contribution to the Flavor of SCJSs

This hydroalcoholic matrix includes a large variety of organic and inorganic compounds that are important in quality evaluation or in fraud control. The study of Franitza et al. allows the classification of 100% of SCJSs and 76% of spirits made from molasses using chemometric analysis [154].

With the aim of preserving the specific features of their manufacturing process while improving product quality, SCJS producers can validate their empirical knowledge using modern analysis techniques. Scientific data can help ensure reproducibility, quality and product authenticity. This chapter presents the chemical compounds identified in cachaça, agricultural rum and grogue. They are divided into two groups according to the production stage at which they are formed. The fermentation congeners group includes organic fatty acids, alcohols, esters, aldehydes and ketones; the maturation-related congeners group contains phenolic and furanic compounds, lactones and terpenoids. And some compounds belong to both groups. 

### 4.1. Fermentation Congeners

#### 4.1.1. The Aliphatic Acids

Organic acids such as myristic, palmitic, acetic or caproic acids are already present in cane juice (Table 3) but most, especially the short-chain fatty acids (<C5), are produced by the yeast metabolism during the alcoholic fermentation [155]. The highest acid, acetic acid (Table 12), comes from the transformation of sugar into acetic acid instead of ethanol in the presence of oxygen during the fermentation [156]. It is also formed following the oxidation of ethanal or from the hydrolysis of acetyl coA and is produced by lactic and acetic bacteria. The second highest, lactic acid is synthesized by *Lactobacillus* while *Bacillus* are responsible for the formation of propionic and butyric acids. 

The production of acids depends on three factors. First, the predominant yeast strain in the wort. For example, yeasts belonging to the genus *Schizosaccharomyces* produce fewer fatty acids than baker’s yeast (*S. cerevisiae*) [20]. The second and third factors are fermentation duration and distillation temperature [8].

The short-chain saturated aliphatic acids, such as formic, acetic, propionic and butyric acids, are associated with unpleasant aromas (pungent, vinegar, milk/butter and sour), whereas the long-chain fatty acids are related to oily, waxy and soapy aromas. They contribute to giving a negative flavor when they are in high concentration in SCJSs. Moreover, they can increase the solubilization of metallic copper during the distillation and thus induce a higher concentration of this contaminant in spirits [8,74]. However, these organic acids have a positive impact on the spirit when they are in small amounts because they are precursors to the formation of esters during distillation and maturation [8,20]. Acetic acid concentration tends to increase during the aging process due he ethanol oxidation [7] or hemicellulose degradation [67]. The slightly acidic pH of SCJSs, around four, can be explained by the presence of these organic acids. 

#### 4.1.2. The Alcohols

Alcohols are the major volatile compounds in unaged spirits (>80%) [54]. With an alcohol strength between 40% and 50% ABV, as in cachaça or agricultural rum, ethanol contributes to the warming and burning perception of the spirit [157]. Among the 26 other alcohols identified in Table 11, isoamyl alcohol, propanol and isobutanol predominate in non-aged and aged SCJS. The higher alcohols, containing more than two carbon atoms, are derived either from amino acids via the Ehrlich catabolic pathway or from pyruvate, which is favored in cases of amino acid deficiency [8,104]. The formation of higher alcohols varies according to the fermentation conditions (strain and amount of yeast, temperature, pH, wort nitrogen content, and duration) [7,8,10,158]. Even if 46% of these alcohols contribute to the fruity aroma and 31% to the sweet flavor, they are also associated with unpleasant aromas such as fusel oil (26%) and solvent (19%). The long-chain fatty alcohols containing up to eight carbon atoms, such as capric and lauric alcohol, are related to waxy/fatty aroma. Depending on their concentration, higher alcohols could be considered as flavoring compounds or contaminants [10]. Hexanol, found also in the raw material, contributes to a herbal aroma, characteristic of freshly distilled SCJS, whereas the presence of 1- and 2-butanol indicates bacterial activity during the alcoholic fermentation [7]. Their concentration is respectively limited in cachaça to 3 and 10 mg/100 mL aa. Glycerol, produced by yeast promotes osmoregulation and redox balance, providing a sweet taste and viscosity [147]. Its content increases in aged SCJSs from the thermal breakdown of wood triglycerides or trans-esterification of triglycerides and ethanol [120]. The concentration of higher alcohols tends to increase during the aging in wooden barrels [67], but it can also decrease because of their oxidation to form aldehydes or esterification with acids [143].

The lowest molecular weight alcohol, methanol, is toxic [8]. For this reason, its concentration is limited to 20 mg/100 mL aa in cachaça and 30 mg/100 mL aa in spirit according to the EU regulation 2019/787 [2]. This compound is produced during the fermentation process by enzymatic demethylation of the methoxy groups of the pectins of the bagasse performed by pectin methylesterase [108]. Filtration, following juice extraction, decreases the fine particles of bagasse in the wort. With a very high perception threshold (1200 mg/L), methanol impacts the flavor negatively with alcoholic and solvent notes [108].

#### 4.1.3. The Aldehydes and Ketones

They originate from the raw material or from the oxidation of fatty acids, alcohols and amino acids [10] and are intermediates to form higher alcohols. The most abundant aldehyde, representing up to 90% of total aldehydes, is ethanal or acetaldehyde (Table 12) [8,108]. This compound, which causes a hangover, derives from the decarboxylation of pyruvate or air-oxidation of ethanol [110,144]. To limit its formation, aeration should be avoided at the end of fermentation [130] and distillation should proceed as soon as possible [108]. This intermediate compound of alcoholic fermentation has a lower boiling point than ethanol and is accumulated in the head fraction during distillation [7]. Hence, ethanal concentration must be below the limitation of 30 mg/100 mL aa for cachaça and 50 mg/100 mL aa for other spirits, according to the EU regulation 2019/787 [2,8].

Aldehydes with less than three carbon atoms, such as acrolein, provide ethereal and pungent aspects to the spirit. This toxic unsaturated aldehyde, produced by bacteria from glycerol and acetaldehyde during fermentation accidents, causes irritation to the skin and mucous membranes and is highly tear-producing. Moreover, it is an intermediate for the acetal, 1,1,3-triethoxy-propane, which is associated with mushroom and solvent aromas. As is the case for ethanal, acrolein is limited to 5 mg/100 mL aa in cachaça. Despite these negative aspects being linked to high concentrations, aldehydes are also known to provide vegetal and fruity aromas (Table 12) [130,159]. Nonanal, already present in the sugarcane juice, and hexanal contribute to green and grass odors [10]. During the aging process, few changes have been observed in the aldehyde concentration, except for aromatic aldehydes, due to the oxidation into acids [67,119], or the formation of acetal from the reaction of ethanal and ethanol in acid medium. 

Regarding ketones, acetone and diacetyl are the highest in terms of concentration. The latter is also produced from pyruvate, as well as ethanal, but under oxidative decarboxylation, by LAB [160]. It is a sub-product in the biosynthesis of amino acids (valine and leucine) with an unpleasant flavor (pungent, buttery aroma).

**Table 12:** Fermentation congeners in SCJS (AR: agricultural rum; C: cachaça; G: Grogue). Concentration range in mg/L or mg/100 mL aa and their sensory descriptor.

*ORGANIC ACIDS*													
Name	N° CAS	Concentration (mg/L)		Type of SCJS	Pungent	Vinegar	Milk/Butter	Rancid/Sour	Sweaty	Oily/Fatty	Waxy	Soapy	References
		Non-Aged	Aged										
***Short-chain aliphatic acids (≤C_5_)***													
**Formic acid**	64-18-6	1.90–4.50	5.9–29.90	C	■								[54,161]
**Acetic acid**	64-19-7	14.60–1211	125–848	C-AR		■							[51,71,122,125,144,145]
**Glycolic acid**	79-14-1	<LOD to 0.16	<LOD to 6.71	C			■						[122,162]
**Propionic acid**	79-09-4	0.0001-0.00396 ^a^	-	C	■	■	■						[54,103,163,164]
**Pyruvic acid**	127-17-3	<LOD to 0.40	<LOD to 3.91	C		■		■					[122,162]
**Lactic acid**	50-21-5	0.32–883	4.89–64.50	C				■					[122]
**Isobutyric acid**	79-31-2	0.43	-	C			■	■	■				[163,164,165]
**Butyric acid**	107-92-6	0.00096–2.30	0.563	C-AR			■	■	■				[54,103,144,163,164,165]
**Succinic acid**	110-15-6	<LOD to 0.05	<LOD to 0.61	C				■					[122,162]
**(S)-2-Methylbutanoic acid**	1730-91-2	-	0.865	AR									[144]
**Isovaleric acid**	503-74-2	-	-	AR					■	■	■		[144]
**Valeric acid**	109-52-4	-	0.89 ^a^	AR				■	■				[54,166]
**2-Methylvaleric acid,**	97-61-0	-	-	C			■	■					[166]
**3-Methylvaleric acid**	105-43-1	-	-	C				■	■				[54]
**Citramalic acid**	597-44-4	<LOD to 0.10	<LOD to 0.80	C									[122]
***Medium-chain saturated fatty acids (C_6_ to C_10_)***													
**Caproic acid**	142-62-1	0.0016–0.0047 ^a^	-	C			■		■	■			[54,103,163,164,166]
**Enanthic acid**	111-14-8	0.00011–0.00093 ^a^	-	C			■						[54,103]
**Caprylic acid**	124-07-2	0.001–0.004	0–0.29	C				■		■			[54,103,144,163,164,167]
**Pelargonic acid**	112-05-0	0.00055–0.00175 ^a^	-	C			■				■		[103,166]
**Capric acid**	334-48-5	0–0.91	0–11.40	C-AR				■			■	■	[54,103,122,144,163,164,167]
***Long-chain saturated fatty acids (>C_10_)***													
**Lauric acid**	143-07-7	0.00032–0.48	0–2.94	C						■			[54,54,103,122,167]
**Myristic acid**	544-63-8	0.01–0.37	<LOD to 5.16	C			■			■	■	■	[54,122]
**Pentadecanoic acid**	1002-84-2	-	-	C							■		[54]
**Palmitic acid**	57-10-3	0.003–0.73	0.10–1.44	C						■	■	■	[54,103,122,164]
**Octadecadienoic acid**	59404-49-8	-	-	C						■			[54,103,122]
**Octadecenoic acid**	2825-79-8	-	-	C						■	■		[54]
***ALDEHYDES & KETONES***													
**Methanol**	67-56-1	1.07–66	0–100	C	■	■							[122,125,152,163,167]
**Propanol**	71-23-8	21.99 ^b^–329	3.76–409	C-G-AR	■		■	■					[10,73,74,89,122,163,164,166]
**Propylene glycol**	57-55-6	0.00008–0.010 ^a^	-	C					■				[103,162]
**Glycerol**	56-81-5	<LOD to 5.30	<LOD to 66	C					■				[127,162]
**1,3-Butanediol**	107-88-0	0.00004–0.00062	-	C					■				[103]
***Saturated fatty alcohols (>C_4_)***													
**1-Butanol**	71-36-3	<LOD to 10.30	0–9	C-AR	■		■						[10,54,122,145,147,164,167]
**Isobutanol**	78-83-1	2.88–256	0–452	C-G-AR		■		■	■				[51,54,81,122,144,166,167,168]
**2-Butanol**	78-92-2	<LOD to 78.20	<LOD to 269	C					■		■		[122,125,162]
**Amyl alcohol**	71-41-0	0.05–0.20 ^c^	-	C	■				■		■		[163,168]
**Isoamyl alcohol**	123-51-3	152–937	450–1290	C-G-AR	■		■		■		■		[51,54,81,122,144,147,163,167,168]
**2-Methyl-1-butanol**	137-32-6	48.02	-	C	■	■	■				■		[48,144,162,163,166]
**(S)-2-Methyl-1-butanol**	1565-80-6	-	245 ^a^	AR		■							[144,162]
**1-Hexanol**	111-27-3	0.00008–9.30	0.644–18.1	C-AR						■	■		[10,103,122,154,163]
**3-Methyl-1-pentanol**	589-35-5	0.00042 ^a^–0.00118 ^a^	-	C			■				■		[103,162]
**Enanthic alcohol**	111-70-6	0.00031–0.00064	-	C		■			■		■		[103,163]
**2-Heptanol**	543-49-7	-	3.38–4.54	AR							■		[10,166]
**Caprylic alcohol**	111-87-5	0.00086–0.00163	8.05–8.46	C-AR			■				■		[10,103,163]
**3-Octanol**	589-98-0	-	0.32–0.35	AR				■		■		■	[10,162]
**4-Octanol**	589-62-8	0.00042 ^a^–0.00373 ^a^	-	C									[103]
**Pelargonic alcohol**	143-08-8	-	3.40–50	AR							■	■	[10,162]
**Capric alcohol**	112-30-1	-	0.018–12.12	AR							■	■	[10,154,162]
**Lauryl alcohol**	112-53-8	0.00045–0.04	4.15–8.67	C-AR								■	[10,103,162,168]
**Myristyl alcohol**	112-72-1	0.16	4.45–7.50	AR							■	■	[10,162,168]
**Cetyl alcohol**	36653-82-4	6.13 ^c^	0.31–1.05	C-AR								■	[10,162,168]
***Unsaturated fatty alcohols***													
***trans*** **-3-Hexen-1-ol**	928-97-2	0.00008–0.0007	-	C									[103,162]
**3-Decen-1-ol**	10339-60-3	-	2.92–3.64	AR						■			[10]
***Saturated aldehydes***													
**Methanal**	50-00-0	0–20.40	2.04–16.70	C									[51,122,130,169]
**Acetaldehyde (ethanal)**	75-07-0	5.7–200	1.55–439	C-AR	■						■	■	[10,51,74,80,89,122,164,165,167,169]
**Propionaldehyde**	123-38-6	0–0.55 ^b^	<LOD to 0.92	C	■	■							[51,122,125,130,169]
**Butyraldehyde**	123-72-8	<LOD to 22	<LOD to 1.34	C						■			[122,169]
**Acetoin**	513-86-0	0.914	-	C				■				■	[163,165]
**Valeraldehyde**	110-62-3	<LOD to 0.31 ^c^	-	C								■	[51,169]
**(R)-2-Methylbutyraldehyde**	33204-48-7	-	0.0189 ^a^	AR									[144]
**(S)-2-Methylbutyraldehyde**	1730-97-8	-	0.0274 ^a^	AR								■	[144]
**Isovaleraldehyde**	590-86-3	<LOD to 0.09	0.233 ^a^	C-AR						■			[51,125,144,169]
**Hexanaldehyde (hexanal)**	66-25-1	<LOD to 0.77	<LOD to 1.07	C-AR					■		■		[10,122,144]
**Heptaldehyde (heptanal)**	111-71-7	-	-	C			■				■		[71,162]
**Caprylaldehyde (octanal)**	124-13-0	-	0.77–0.93	AR			■				■	■	[10,145,165]
**Pelargonaldehyde (nonanal)**	124-19-6	-	2.44–2.72	AR					■				[10]
**Capraldehyde (decanal)**	112-31-2	-	0.68–0.87	AR								■	[10]
***Unsaturated aldehydes***													
**Acrolein/acrylaldehyde**	107-02-8	0–5.80 ^b^	<LOD to 0.36	C		■							[122,130,169]
**Crotonaldehyde**	123-73-9	<LOQ to 5.32 ^c^	-	C									[51]
**2,4-Nonadienal**	5910-87-2	-	-	C					■		■	■	[71]
***trans*** **-2-Nonenal**	18829-56-6	-	-	C					■				[71]
***trans*** **-*trans*-2,4-Decadienal**	25152-84-5	-	0.00115 ^a^	AR					■		■	■	[144]
***Ketones***													
**Acetone**	67-64-1	0–23	<LOD to 6.90	C		■	■					■	[122,125,130,167]
**Diacetyl**	431-03-8	0–9.77	0.0437	C-AR		■							[71,125,130,144,145,167,170]
**Cyclopentanone**	120-92-3	0–2.87	-	C									[51,167]
**1-Octen-3-one**	4312-99-6	-	-	C				■			■		[71,162]
**2-Nonanone**	821-55-6	0–0.00028 ^a^	0.87–2.55	C-AR					■		■	■	[10,103]
**2-Undecanone**	112-12-9	-	0.94–1.72	AR					■			■	[10]
**2-Pentadecanone**	2345-28-0	-	0.97–2.14	AR									[10]
***Acetals***													
**Acetal**	105-57-7	-	25 ^a^	AR			■				■	■	[71,144,164,171]
**1,1,3-Triethoxypropane**	7789-92-6	-	-	C			■						[172,173]
***ESTERS***													
**Ethyl formate**	109-94-4	-	-	C	■			■	■				[54]
**Methyl propionate**	554-12-1	0.08	-	C	■	■							[162,168]
**Ethyl acetate**	141-78-6	1.56 ^c^–623 ^c^	15.5–1160	C-AR	■	■	■						[10,51,54,74,80,122,163,167,168,169]
**Ethyl acrylate**	140-88-5	-	-	C	■								[71]
**Ethyl lactate**	97-64-3	<LOD to 75.90	2.21–120	C-AR	■	■	■						[10,122,125,130]
**Ethyl isobutyrate**	97-62-1	-	-	C		■	■						[71,171]
**Ethyl butyrate**	105-54-4	<LOD to 19.70 ^c^	<LOD to 3.20	C-AR		■	■						[54,71,122,144,163,171,174]
**Isoamyl acetate**	123-92-2	-	1.06 ^a^–13.83	AR	■	■	■						[10,54,71,144,163,166,171]
**Ethyl valerate**	539-82-2	-	0.0515–2.02	AR		■							[10,144]
**Ethyl isovalerate**	108-64-5	-	0.211 ^a^–2.56	AR		■	■						[10,144,154,166]
**Ethyl 2-methylbutyrate**	7452-79-1	-	-	C-AR		■							[71,144,171]
**Ethyl(S)-2-methylbutanoate**	10307-61-6	-	0.23	AR		■							[144]
**Propyl butyrate**	105-66-8	0.08	-	C		■	■						[162,168]
**Ethyl caproate**	123-66-0	<LOD to 9.84 ^b^	0.55–66.42	C-AR		■	■	■					[10,54,103,122,130,144,163,164,171]
**Diethyl succinate**	123-25-1	0.00308–0.0069	-	C						■		■	[103,164]
**Amyl propionate**	624-54-4	0.02 ^c^	-	C		■	■						[168]
**2-Hexyl acetate**	5953-49-1	-	-	C		■							[54,162]
**Ethyl enanthate**	106-30-9	0.05 ^c^	3.29–4.07	C-AR						■			[10,162,168]
**Ethyl cyclohexanoate**	3289-28-9	-	0.00103 ^a^	AR		■							[144,162]
**Ethyl caprylate**	106-32-1	0–16.28	586.18–723.19	C-AR							■		[10,54,103,130,163,164,171,174]
**Isoamyl valerate**	2050-09-1	0.01 ^c^	-	C		■							[162,168]
**Ethyl pelargonate**	123-29-5	0.1508–0.552	15.47–21.45	C-AR		■			■		■		[10,166,174]
**Methyl caprate**	110-42-9	-	0.00004–0.00033 ^a^	AR		■			■	■			[154]
**Ethyl caprate**	110-38-3	0.132 ^c^–13.01 ^c^	-	C		■	■	■			■		[10,54,130,170,171,174]
**Isoamyl octanoate**	2035-99-6	0.0666 ^c^–0.234 ^c^	-	C		■	■			■		■	[162,174]
**Ethyl laurate**	106-33-2	0.015 ^a^–4.03 ^c^	-	C			■	■	■		■	■	[10,54,103,174]
**Ethyl myristate**	124-06-1	0.00042 ^a^–0.00706 ^a^	-	C			■				■		[103,162]
**Ethyl palmitate**	628-97-7	-	-	C						■			[54,162]
**Ethyl 9-hexadecenoate**	54546-22-4	0.00088–0.00175 ^a^	-	C									[103]
**Methyl linoleate**	112-63-0	-	-	C						■			[54,162]
**Ethyl margarate**	14010-23-2	-	-	C									[54]
**Ethyl linolate**	544-35-4	-	-	C		■				■			[54,162]
**Ethyl stearate**	111-61-5	-	-	C							■		[54,162]

^a^ Value in μg/L in the reference and converted in mg/L, ^b^ value in mg/100 mL aa and converted in mg/L with alcohol strength indicated in the article, ^c^ value in mg/100 mL aa, - value not found in the literature.

#### 4.1.4. The Esters

Esters are key compounds in fermentation congeners because a correlation has been established between their content and the aromatic quality of spirits. They have a strong odorant power due to a low olfactory perception threshold [108]. According to the Brazilian legislation, cachaça must contain less than 200 mg/100 mL aa esters expressed in ethyl acetate, whereas this limit is fixed to 130 mg/100 mL aa in the EU [2,8]. These compounds are produced either through the condensation of acetyl CoA with ethanol (acetate esters) or through the condensation of a fatty acid with ethanol (acyl esters) [104]. Thus, ethyl esters of fatty acids and acetates of higher alcohols are produced during alcoholic fermentation by enzymatic reaction by yeasts [117] but also by lactic bacteria during malolactic fermentation [160]. The ester rate depends on three elements: the variety of cane, the fermentation and distillation conditions [20]. First, cane wax is a source of fatty acids that favors the content of ethyl esters. Secondly, the oxygen availability and the yeast strain impact esters formation whereas high temperature negatively affects their formation during the fermentation [20,54]. Third, the ethyl acetate concentration is different according to which distillation apparatus is used (pot or column still) [108]. Esters are known to bring a fruity and sweet flavor to spirits (Table 12). Ethyl acetate, the predominant ester in distillate (80% of total esters) [8,143], contributes to a pleasant flavor at low concentrations. On the contrary, an important amount in the spirit, due to an unwanted acetic bacteria activity [108], has a sensory negative impact (solvent, acidic). Esters most favorable to aromatic richness (‘bouquet’) are those with middle and long-chain fatty acids (≥C_5_). From 8 carbon atoms, esters contribute to fatty/oily aromas whereas the fatty acid esters with at least 10 carbon atoms give waxy or soap notes. During maturation in wooden barrels, their concentration increases thanks to esterification reactions between alcohols and fatty acids [143]. According to studies on cachaça, esters concentration depends on wood species. Oak is the one providing the highest content after 6 months [119] or 3 years [143]. 

### 4.2. Maturation-Related Congeners

Phenolic compounds bring an important contribution to the sensory profile of aged spirit (Table 13). This chemical family contains one or several hydroxylated benzene rings [128]. Some of them are already found in the sugarcane juice such as benzaldehyde, eugenol or 4-vinylguaiacol (Table 4). As a result of all physical and chemical reactions occurring during the aging process (Table 10), the spirit acquires color, new aromas and roundness making its organoleptic profile more harmonious. The aged spirit color ranges from light yellow to dark brown depending, for the most, on the duration of storage in the barrel, the number of uses [74] and the wood species [143] thanks to both phenolic and furanic compounds extraction [67,118,123]. Alike the color, texture evolves during the aging as gallic acid, sugars and glycerol concentration increase [10,67]. Besides, mouthfeel and taste, crucial for consumer acceptance, can be pleasant with the sweetness. On the other hand, they can be unpleasant because of astringency due to high content of tannins and its derivative (gallic acid) [117]. However, oxidation and polymerization of tannins during the wooden aging lead to a reduction in the astringency strength [175]. The tannin family is divided into two groups: hydrolyzable tannins (ellagitanins, gallotanins) or condensed tannins (catechin, gallocatechin) [175,176]. The second group is at the origin of flavonoids, which are odorless. With a molecular structure rich in alcohol function (OH) and double carbon bonds (C=C), these phenolic compounds have a more or less antioxidant capacity. (+)-Catechin seems to possess a better efficient antioxidant activity than quercetin and kaempferol [142]. 

While fresh distillate features freshness, fruity, floral, and green aromas, the wooden aged distillate is more widely defined by woody, spicy and empyreumatic notes (Table 13). Among the simple phenolic compounds (one aromatic ring) some are noteworthy for their contribution to sensory profile. Eugenol, as well as its precursor, guaiacol, brings spicy (clove) and smoky aromas with low olfactory thresholds [128]. The other lignin-derivative, guaiacol- (vanillin, vanillic acid, coniferaldehyde) and syringol- (sinapaldehyde, syringic acid and syringaldehyde) compounds are widely studied because they are aging markers [129]. The study of Castro et al. indicates that American oak barrel (*Q. alba*) releases in cachaça a higher amount of benzoic aldehydes (syringaldehyde and vanillin) and benzoic acids (syringic acid and vanillic acid) compared to French oak barrels (*Q. petraea*) [129]. Conversely, Madrera et al. found in brandy a higher content of these compounds in French oak (*Q. sessilis*) [177]. It is relevant to note that the aging environment conditions were certainly different (Europe/Brazil). 

The γ-lactones, so-called oak or whiskey lactones, derive from the oxidation of lipids by LAB during lactic fermentation and from glycosidic compounds during the heat treatment [117,175]. These volatile compounds are key odorants in aged spirits bringing coconut aroma (Table 13). Among the two isomeric *cis*- and *trans*-forms of oak lactones, the first contributes the most because of its low perception threshold [117]. 

Coumarin and scopoletin are considered markers of aging because these compounds are found in the spirit after a long period of storage in a barrel [178]. However, the concentration of coumarin ought to be monitored because of its toxicity [123] considering that American oak (*Q. alba*) provides a higher scopoletin amount than French oak (*Q. sessillis*) in the case of brandy [115,177].

The thermal degradation of hemicellulose from the wood barrel induces the formation of furanic aldehydes, such as 5-HMF and furfural. Slightly found in unaged spirits except when the juice comes from burned cane [119], the sum of furfural and 5-HMF is limited to 5 mg/100 mL aa in cachaça. These C_4_ heterocyclic-based compounds are formed by the dehydration of pentoses caused by high temperatures under acidic conditions and/or by the Maillard reaction during the barrel-making process [67]. These compounds bring toasted, woody and toasted almond aromas to the spirit and a peculiar color as mentioned below (Table 13) [67]. 

Terpenes and terpenoids or isoprenoids, are a large group of organic compounds found in plants. Terpenoids include acyclic (e.g., citronellol, linalool), cyclic (e.g., menthol) compounds, composed of isoprene unit (C5), and C13-carotenoid-derived compounds, called norisoprenoids (e.g., ionone and damascenone) (Table 14). Their concentration in unaged spirits depends on the cane variety [10]. Some free or glycosylated odorless hydrocarbons, already present in the raw material, may be transformed during the distillation process [179]. During the maturation process, their formation can be related to the thermal or oxidative degradation of carotenoids [10] and their presence in aged distillate varies depending on the wood species [119]. The most abundant terpene, d-limonene, already present in the raw material is associated with floral aroma. The C13-norisoprenoids provide fruity notes; and sesquiterpenes (C15) contribute to a woody aroma. Terpenoids provide a pleasant aroma to spirits (floral, citrus and vegetal) [54,145]. β-ionone is a marker used to differentiate SCJS from those made from molasses [154].

**Table 13:** Aromatic compounds in SCJS (AR: agricultural rum; C: cachaça). Concentration range in mg/L or mg/100 mL aa and their sensory descriptor.

	N° CAS	Concentration (mg/L)		Type of SCJS	Fruity	Sweet	Woody	Phenol	Balsamic	Floral	Spicy	Empyreu ^1^	Chemical	References
		Non-Aged	Aged											
***Phenolic compounds and derivatives***														
Phenol	108-95-2	0.0005 ^a^–0.0041 ^a^	0.0002 ^a^–0.014 ^a^	C				■						[142,162]
*o*-Cresol	95-48-7	0.001 ^a^–0.005 ^a^	0–0.097 ^a^	C				■					■	[142,162]
*p*-Cresol	106-44-5	-	0.00341 ^a^	AR				■					■	[144,162]
Guaiacol	90-05-1	0.0002–0.003	0.0005–0.047	C-AR				■				■	■	[54,71,103,142,144,171]
Protocatechuic acid	99-50-3	-	2.65–2.83	AR				■	■					[10,162]
Gallic acid	149-91-7	-	0-12.97	C-AR										[10,123,167]
2-Ethylphenol	90-00-6	0–0.0027 ^a^	0.0009 ^a^–0.124 ^a^	C				■						[142,162]
3,5-Xylenol	108-68-9	0–0.0058 ^a^	0–0.065 ^a^	C				■	■			■	■	[142,162,163]
4-Ethylphenol	123-07-9	0–0.021 ^a^	0.0023 ^a^–0.117 ^a^	C-AR				■				■		[142,144,162]
Vanillin	121-33-5	-	0–3.29	C-AR		■		■			■			[10,54,71,144,144,162,167,171]
Syringol	91-10-1	-	-	C		■	■	■	■			■	■	[54,162]
Vanillic acid	121-34-6	-	0–2.24	C-AR		■		■			■			[10,54,162,167]
4-Vinylguaiacol	7786-61-0	-	-	C			■	■			■	■		[54,71,162]
4-Ethylguaiacol	2785-89-9	0.005 ^a^–0.052 ^a^	0.0006 ^a^–0.298 ^a^	C-AR		■		■			■	■		[142,144,162]
*p*-Coumaric acid	7400-08-0	-	0–7.09	C-AR										[10,167]
Caffeic acid	331-39-5	-	0–1.39	AR										[10]
Syringaldehyde	134-96-3	-	0–6.11	C-AR		■	■					■		[10,162,167]
Syringic acid	530-57-4	-	0–4.57	C-AR										[10,167]
Anethole	104-46-1	-	12.62–17.61	AR		■							■	[10,162]
Eugenol	97-53-0	0.0012–0.088	0.002–0.1183	C-AR			■				■	■		[71,142,144,162,171]
Dihydroeugenol	2785-87-7	-	0.0193	AR		■		■			■	■		[144,144,162,171]
Coniferaldehyde	458-36-6	-	0–0.25	C							■	■		[162,167]
Scopoletin	92-61-5	0–0.030	0–1.31	C										[142]
Methoxyeugenol	6627-88-9	-	-	C	■		■	■			■			[54,162]
Sinapaldehyde	4206-58-0	-	0–1.20	C										[167]
Ellagic acid	476-66-4	-	0–4.51	C-AR										[10,167]
***Flavonoids***														
Kaempferol	520-18-3	0	0–1.95	AR										[10,142]
Epicatechin	490-46-0	0–0.22	0–1.53	C										[142,167]
Catechin	154-23-4	0–0.15	0–0.42	C										[142]
Quercetin	117-39-5	0	0–2.30	C-AR										[10,142]
Myricetin	529-44-2	-	0–3.48	AR										[10]
Rutin	153-18-4	-	4.34–7.99	AR										[10]
***Aromatic alcohols and acids***														
Benzyl alcohol	100-51-6	-	-	C		■		■		■			■	[54,162]
Benzoic acid	65-85-0	-	-	C					■				■	[54,162]
Phenethyl alcohol	60-12-8	0.02 ^c^	6.66–17.10	C-AR		■				■				[10,54,71,144,163,168,171]
Cinnamic alcohol	104-54-1	6.95 ^c^	-	C		■			■	■	■			[168]
***Aromatic esters***														
Phenyl acetate	122-79-2	0–0.00014	-	C			■	■				■	■	[103,162]
Ethyl benzoate	93-89-0	1.84	-	C	■	■							■	[162,168]
Phenethyl acetate	103-45-7	0.00016 ^a^–0.00029 ^a^	-	C	■	■								[71,103,162,163,164,171]
Ethyl hydrocinnamate	2021-28-5	-	0.00354 ^a^	AR	■					■				[144,162]
***Lactones***														
β-Angelica lactone	591-11-7	-	-	C		■								[54]
Sotolon	28664-35-9	-	0.0162	AR		■						■		[144,162]
Coumarin	91-64-5	-	-	C		■						■		[54,162]
Hydrocoumarin	119-84-6	-	-	C		■			■			■		[54,162]
*cis*-Whiskey lactone	80041-00-5	-	1.12	AR	■		■							[144,171]
*trans*-Whiskey lactone	80041-01-6	-	0.124	AR	■		■							[144,171]
γ-Nonalactone	104-61-0	-	-	AR	■									[144,171]
γ-Dodecalactone	2305-05-7	-	0.0037–0.0070	AR	■	■								[154,162]
***Aromatic aldehydes and ketones***														
Benzoic aldehyde	100-52-7	0–8.57	1.77–8.61	C	■	■	■					■		[10,51,122,130,147,162,169]
Acetophenone	98-86-2	0–2.90	-	C	■	■				■			■	[51,167]
***Furanic compounds***														
3-Furaldehyde	498-60-2	-	0.00108–0.0128 ^a^	AR										[154]
Furfural (2-Furaldehyde)	98-01-1	0–4.57 ^c^	1.12 ^b^–24.59 ^b^	C-AR										[10,51,67,130,163,169,170,180,181]
Furfuryl alcohol	98-00-0	0-1.30	-	C										[103,162]
5-HMF	67-47-0	<LOQ to 7.23 ^c^	0.8–19.40	C-AR										[10,51,67,125,162,169,170]
Furfuryl acetate	623-17-6	0.00029 ^a^–0.00105 ^a^	-	C										[103,162]
***Aromatic hydrocarbons***														
*o*-Xylene	95-47-6	-	0.71–1.68	AR										[10]
*p*-Cymene	99-87-6	-	14.7–18.25	AR									■	[10]
Cumene	98-82-8	-	2.12–3.93	AR									■	[10]
TDN ^2^	30364-38-6	-	3.09–4.32	AR										[10]

^1^ Empyreumatic aromas; ^2^ 1,1,6-Trimethyl-1,2-dihydronaphthalene, ^a^ value in μg/L in the reference and converted in mg/L, ^b^ value in mg/100 mL aa and converted in mg/L with alcohol strength indicated in the article, ^c^ value in mg/100 mL aa, - value not found in the literature.

### 4.3. The Other Organic Compounds

Acetate, the anion of acetic acid, was quantified by IC in cachaça and grogue by Pereira et al. (Table 15). It is a precursor of esters and acids [80]. 

The sulfur compounds in spirit are known to degrade the sensory profile (sulfurous flavor) [162]. The major sulfur compound, dimethylsulfide, is probably generated from the degradation of sulfur-containing amino acids or from the reduction of sulfate salts in the wort during fermentation [20,182]. These compounds form insoluble copper salts during the distillation, which are found in the vinasse [117].

A noteworthy ester is ethyl carbamate (EC), also known as urethane, with no sensory contribution but with potentially carcinogenic properties [183]. Countries such as Canada and Brazil restrict the maximum acceptable concentration of EC to, respectively, 150 μg/L and 210 μg/L in spirits, whereas, in Europe, a recommendation from the European Commission encourages producers to limit this concentration in stone fruit spirits (e.g., kirsch, plum spirits, apricot spirits, etc.) to 1000 μg/L [8,159]. 

Many factors can promote EC formation throughout the SCJS-making process. A better control of the fermentation (temperature, nitrogen supply) and distillation (reflux system, wine feed rate and temperature) parameters could avoid the formation of EC precursors, namely, hydrocyanic acid [7,184]. For cachaça, the double distillation helps to reduce the concentration of EC. However, in the case of Rhum Agricole AOC from Martinique, this practice is prohibited [185]. 

### 4.4. Inorganic Compounds

The level of inorganic compounds has not been investigated for agricultural rums (Table 16). Nevertheless, these chemical species are important for the quality of the spirit and its flavor [80,117]. Found in sugarcane juice, depending on the variety, they are essential for yeast fermentation. During the distillation step, their concentration varies in the distillate according to the inner materials used in a column or pot still. 

Copper, extracted into the spirit from the still, has a positive action on the aroma because it reduces the unpleasant taste due to sulfur compounds [184]. However, it is considered an environmental pollutant when it is found in high concentration in the vinasse. Moreover, copper catalyzes the formation of EC from cyanide, [67]. So, producers try to reduce its concentration in fresh distillate.

## 5. Conclusions

The family of SCJSs is a diverse category presenting styles that are the reflection of the terroir, tradition and expertise acquired over generations. Economic sectors related to this spirit are currently developing, boosted by spiritourism. Producers strive to improve the quality thanks to the sensory and chemical characterization of their spirits. The current scientific data available on most of these SCJS are incomplete. For example, the specificities of aging under tropical conditions are less commonly studied than those under temperate climates. Nevertheless, additional data are required to enhance the process and help develop new outstanding products. Hence, investments in improving quality will lead to more innovation and optimization and finally open the way to premiumization. 

Some producers have put forward unique production methods, such as organic farming, mono-variety production, eco-friendly practices (use of recycled bottles, packaging), finishing in barrels that have contained other alcoholic beverages (wine, Porto, whiskey, etc.), and aging with woods other than oak. 

Altogether, the future of SCJSs seems promising, especially if producers succeed in preserving their distinctive features while facing new challenges. In addition to improving yield and quality, these industries are concerned with reducing their carbon footprint and impact on fauna and flora (discharge of vinasse, cultivation practices).

## Figures and Tables

**Table 1:** Some well-known spirits in the world, the principal raw material used and the main region of production.

Spirit	Raw Material	Main Producer Countries
Armagnac (AOC)	White wine	France
Baijiu	Sorghum, rice or cereal	China
Brandy	Wine	Spain, Italy, Portugal, Greece
Cachaça	Sugarcane juice	Brazil
Cognac (AOC)	Wine	France
Mezcal	Agave	Mexico
Pisco	Wine	South America (Chili; Peru)
Rhum Agricole	Sugarcane juice	French West Indies
Rum	Sugarcane and its derivatives	Caribbean, South America
Shōchū	Barley, rice, potato	Japan
Schnaps	Potato or cereal + Fruit	Germany
Soju	Cereal, potato	South Korea
Tequila	Blue agave (*Agave tequilana*)	Mexico (State of Jalisco)
Vodka	Cereal, potato, fruit	Russia and Scandinavia
Whiskey	Cereal	Scotland, USA, Canada

**Table 2:** Sugarcane classification [16], chromosome number of the six species of genus *Saccharum* [14,17,18].

Classification				
Order	Poales	Species		
Family	Poaceae	Wild cane	*S. spontaneum* *S. robustum*	2n = 40 to 128 2n = 60 to 200
Subfamily	Panicoideae	Noble cane	*S. officinarum*	2n = 80
Tribe	Andropogoneae	Chinese cane	*S. sinense*	2n = 116 to 120
Sub-tribe	Saccharinae	Indian cane	*S. barberi*	2n = 81 to 124
Genus	Saccharum	Edible cane	*S. edule*	2n = 60 to 122

**Table 3:** Chemical properties and composition of sugarcane.

	Chemical Properties and Composition	References
Color	Light gray to dark green	[36]
pH	4.9–5.5	[36]
Sugars	Sucrose, glucose, fructose	[19,20]
Organic acids	*trans*-/*cis*-aconitic, malic, oxalic, citric, d-gluconic, succinic, l(+)-lactic acids	[32]
Nitrogenous compounds	Amino acids, proteins	[36]
Organic compounds	Starch, gums, chlorophyll, anthocyanin, biotin, polysaccharides	[36]
Fatty compounds	Waxes, fatty acids, phosphatides, sterols	[37,38]
Phenolic acids	Chlorogenic, cinnamic, hydroxycinnamic, sinapic and caffeic acids	[38]
Phenolic compounds	Tannin, flavones (tricin, apigenin, luteolin and glycosides derivatives)	[38]
Inorganic compound	K, Cl, Mg, P, S, Ca, Si, Fe, Na, Al, Mn, Zn; SiO_2_, K_2_O, P_2_O_5_, Fe_2_O_3_	[36,37]
Vitamins	A, C, B1, B2, B3, B5, and B6	[35,36]

**Table 4:** Volatile aroma compounds identified in sugarcane and its juice [39]. nd: not detected.

Volatile Organic Compounds	N° CAS	Odor Description	Sugarcane	Juice
**Acids**				
Acetic acid	64-19-7	Sour	X	X
Isobutyric acid	79-31-2	Rancid, butter, cheese	X	X
Caproic acid	142-62-1	Sweat	X	X
Myristic acid	544-63-8	Sweet spicy	X	X
Pentadecanoic acid	1002-84-2	Waxy	X	X
Palmitic acid	57-10-3	Slightly waxy, fatty	X	X
**Alcohols**				
2,3-Butanediol	513-85-9	Fruit, onion	X	X
*cis*-2-Penten-1-ol	1576-95-0	Green, plastic, rubber	nd	X
1-Hexanol	111-27-3	Resin, flower, green	X	X
*cis*-3-Hexen-1-ol	928-96-1	Grass	X	X
2-Heptanol	543-49-7	Mushroom	X	X
Phenylethyl alcohol	60-12-8	Honey, spice, rose, lilac	X	X
1-Octen-3-ol	3391-86-4	Mushroom	X	X
2-Ethyl-1-hexanol	104-76-7	Rose, green	X	X
Caprylic alcohol	111-87-5	Chemical, metal, burnt	nd	X
Lauryl alcohol	112-53-8	Fat, wax	nd	X
**Aldehydes**				
Benzaldehyde	100-52-7	Almond, burnt, sugar	X	X
2,4-Heptadienal	4313-03-5	Nut, fat	X	X
***trans*** -2-Octenal	2548-87-0	Green, nut, fat	X	X
***trans*** -2-Nonenal	18829-56-6	Cucumber, fat, green	nd	X
Nonanal	124-19-6	Fat, citrus, green	X	X
**Esters**				
Ethyl palmitate	628-97-7	Wax	nd	X
Dibutyl phthalate	84-74-2	Faint odor	X	X
**Hydrocarbons**				
Styrene	100-42-5	Balsamic, gasoline	X	X
*m*-Xylene	108-38-3	Plastic	X	X
d-Limonene	5989-27-5	Citrus, mint	nd	X
**Phenols**				
4-Vinylguaiacol	7786-61-0	Clove, curry	X	X
Eugenol	97-53-0	Clove, honey	X	X
2,4-Di-*tert*-butylphenol	96-76-4	Phenolic	X	X
2,6-Di-*tert*-butyl-methylphenol	128-37-0	Mild phenolic camphor	X	X
**Heterocyclic compound**				
2-Pentylfuran	3777-69-3	Green bean, butter	X	X

**Table 5:** Rhum Agricole AOC Martinique specifications according to the decree of 31 December 2020 [64]. HPA: Hectoliter pure alcohol.

Step of Process		AOC Martinique Technical Specifications
**Territory**	Geographical area	Delimited on 23 municipalities of Martinique
**Raw material**	Cane species	*Saccharum officinarum* and *S. spontaneum* hybrids Cane varieties approved by INAO
	Harvest period	From January 1st to August 31st
	Culture	Yield ≤ 120 t/acres
	Juice quality	Brix ≥ 12% and pH ≥ 4.7
**Juice extraction**	Process	Cold mechanical extraction with water and/or juice from the last roll. The juice must be filtered
**Fermentation**	Type	Discontinuous in an open vat of 500 hL max
	Yeast	*Saccharomyces*
	Duration	<120 h
	Wine alcohol content	≤7.5% ABV
**Distillation**	Type	Multi-stage continuous distillation column without reflux, ‘creole’ column
	Stripping section	15 trays in stainless steel or copper—diameter: 0.7 to 2 m
	Rectifying section	5 to 9 trays in copper
	Rum alcohol content	65 to 75% ABV
	Non-ethanol content (NEC)	≥225 g/HPA
**Rums**	White rum	Storage at least 6 weeks in stainless steel vat—NEC ≥ 225 g/HPA
	Straw rum (‘Élevé-sous-bois’)	At least 12 months in an oak vessel and NEC ≥ 250 g/HPA
	Aged rum	At least 3 years in an oak vessel and NEC ≥ 325 g/HPA
**Final validation**	Each batch is tasted by a panel of experts who approved the AOC Martinique sensory profile	

**Table 6:** Limits for compounds in cachaça according to Brazilian legislation [8].

Component	Limit Value	Unit
**Alcohol strength**	38–48	% of ethanol (*v*/*v*) at 20 °C
**Volatile acidity (expressed in acetic acid)**	150	mg/100 mL anhydrous alcohol
Methanol *	20	
**Total higher alcohols**	<360	
1-butanol *	3	
2-butanol *	10	
**Total esters (ethyl acetate)**	200	
Ethyl carbamate *	210	μg/L
**Total aldehydes (acetaldehyde)**	30	mg/100 mL anhydrous alcohol
Acrolein *	5	
**Furfural + 5HMF**	5	
Lead *	200	μg/L
Arsenic *	100	
Copper *	5	mg/L

* Contaminants.

**Table 7:** Proposed differentiation of SCJS according to their specific manufacturing processes. Dashed arrow: optional.

SCJS	Main Production Area	Harvest/Milling	Fermentation	Distillation	Aging
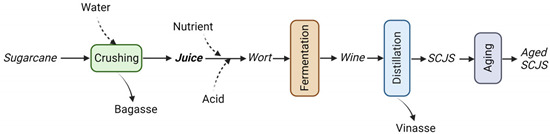					
**Aguardiente de Cana**	Central and South America	Traditional method	Spontaneous for small-size producers	Pot still	Oak or tropical wood
**Cachaça**	Brazil	From May to November	Spontaneous or with adding yeast. Continuously open vessel	Simple or double distillation Pot or column still	At least 12 months in oak or tropical wood
**Clairin**	Haiti	Mainly manual harvesting	Addition of aromatic plants	Mostly copper Charentais alembic	Wooden barrel
**Grogue**	Cape Verde	From January to July with ‘trapiche’: equipment powered by oxen or mules	Long fermentation: days to weeks Spontaneous or addition of yeast with sugar 6 g/L	Artisanal pot still	At least 12 months in wood container
**Agricultural rum**	French overseas departments and Madeira	Imbibition water or composed juice	Imbibition water or composed juice	Pot or column still	Oak barrel
**Rhum Agricole AOC** **Martinique**	Martinique	From January to August. Extraction at ambient temperature	Open vat Only *Saccharomyces genus* <5 days	Simple, creole column still From January to August	Only oak barrel Addition of caramel prohibited

**Table 8:** Wort physicochemical parameters influencing fermentation efficiency.

Condition	Low	High
**pH**	↓ Yeast activity	↑ Bacteria proliferation
**Temperature**	↓ Membrane fluidity → sluggish fermentation	↓ Yeast activity
**Oxygen**	↓ Membrane permeability	↑ ROS production
**Nutrient (nitrogen)**	Sluggish fermentation	↑ Higher alcohol and ester concentration

**Table 11:** Descriptors used in SCJS sensory analysis for new distillate or mature spirits.

Type of Aroma	Descriptor	Reference Standard	New Distillate Descriptors	Mature Descriptors	References
Fruity	Fruity in general	Isoamyl acetate Ethyl caprylate	X	X	[71]
	Apple	Ethyl caproate	X		[71]
	Melon	Dimethylheptanal	X		[71]
	Banana	Banana aroma	X	X	[150]
Floral	Honey	Natural honey	X	X	[95,150]
	Floral/rose	2-Phenylethanol	X		[71]
	Sugarcane	Sugarcane	X		[152]
Herbal	Grassy/Vegetable	*cis*-3-Hexen-1-ol	X		[46,71,95,122]
Spicy	Vanilla	Ethyl vanillin/vanilla	X	X	[95,146,150]
	Clove	Eugenol/Cloves aroma		X	[71,150]
Animal	Leathery	-		X	[95]
Wood	Woody/Oak	Oak wood chips		X	[71,95,146,150]
	Phenolic	-		X	[95]
Empyreumatic	Burntsnug	-		X	[95]
	Caramel	Maltol-Caramel syrup		X	[71,95,146]
	Smoky	Guaiacol-Smoked bacon		X	[71,146]
	Coffee	Dark roast coffee		X	[146]
Default	Oily	Heptanal	X	X	[71,95]
	Sulphury	Dimethyl sulfide	X		[71,95]
	Buttery	Diacetyl	X		[71,95]
	Soapy	Ethyl laurate	X		[71,95]
	Mouldy	2,4,6-Trichloroanisole	X		[71,95]
	Solvent	2-Methylpropan-1-ol	X		[71,95]
	Vinegar	Acetic acid	X	X	[71]
	Medicine	Thymol	X	X	[71]
	Alcoholic	Ethanol	X	X	[150]
	Fermented	Compressed yeast	X		[150,152]
	Bagasse	Bagasse	X		[152]
Trigeminal sensation	Pungent	Formic acid	X	X	[71,95]
	Astringent	Over-brewed green tea	X	X	[146]
	Sweetness	Sugar	X	X	[95,122]
	Bitter	Caffeine solution	X	X	[122,150]
	Burning	-	X	X	[152]
	Sour taste	Acetic acid	X	X	[153]
Retronasal	Persistence	-	X	X	[149]

**Table 14:** Terpenes and terpenoids in SCJS (AR: agricultural rum; C: cachaça). Concentration range in mg/L or mg/100 mL aa and their sensory descriptor.

Name	N° CAS	Concentration (mg/L)		Type of SCJS	Resinous	Terpenoid	Vegetal	Floral	Citrus	Fruity	Woody	Menthol	References
		Non-Aged	Aged										
***Terpenes***													
d-Limonene	5989-27-5	-	89.51–99.65	AR				■					[10]
Sesquisabinene	58319-04-3	-	-	C							■		[54]
***Terpenoids***													
Citronellol	106-22-9	0.00113–0.00292	-	C			■						[103]
Geraniol	106-24-1	0.00015 ^a^–0.21 ^c^	-	C				■	■				[103,168]
Linalool	78-70-6	0.00035 ^a^–0.00138 ^a^	-	C		■		■	■				[103]
Menthol	1490-04-6	0.095 ^a^–0.73 ^c^	6.85–8.02	C-AR								■	[10,103,168]
α-Terpineol	98-55-5	0.00018 ^a^–0.00046 ^a^	-	C	■	■			■				[103,162]
d-Isomenthol	23283-97-8	-	1.44–1.77	AR			■					■	[10,162]
Farnesol	106-28-5	-	1.14–2.09	AR			■	■					[10]
Nerolidol	7212-44-4	-	-	C				■			■		[54,119]
α-Cadinol	481-34-5	-	-	C					■		■		[54]
α-Elemol	639-99-6	-	-	C		■			■		■		[54]
Torulosol	1438-65-9	-	-	C			■						[54]
***C13-norisoprenoids***													
β-Ionone	79-77-6	-	0.0061–0.012	AR				■		■			[115,162]
β-Damascenone	23696-85-7	-	0.0437–3.31	AR				■		■	■		[10,144,162]

^a^ value in μg/L in the reference and converted in mg/L, ^c^ value in mg/100 mL aa, - value not found in the literature.

**Table 15:** Other organic compounds in SCJS (C: cachaça; G: grogue). Concentration in mg/L.

Compound Name	N° CAS	Concentration (mg/L)		Type of SCJS	References
		Non-Aged	Aged		
Acetate	71-50-1	<LOD to 2.93	-	C-G	[80]
Dimethylsulphide	75-18-3	<LOQ to 52.10 ^c^	0.01–0.59	C	[51,122]
2,3-Butanedione monoxime	57-71-6	<LOQ to 0.859 ^c^	-	C	[51]
Ethyl carbamate	51-79-6	0–1455.23 ^c^	<LOD to 0.138 ^a^	C	[51,74,122,170,180,181]
Methyl anthranilate	134-20-3	-	-	C	[54,162]

^a^ Value in μg/L in the reference and converted in mg/L, ^c^ value in mg/100 mL aa, - value not found in the literature.

**Table 16:** Inorganic compounds in SCJS (C: cachaça; G: grogue). Concentration in mg/L.

Name	Molecular Formula	N° CAS	Concentration (mg/L)		Type of SCJS	References
			Non-Aged	Aged		
***Anions***						
Bicarbonate	HCO_3_^−^	71-52-3	<LOD to 105.15	-	C	[80]
Chloride	Cl^−^	16887-00-6	<LOD to 183	-	C-G	[80]
Sulfate	SO_4_^2−^	14808-79-8	<LOD to 12.03	-	C-G	[80]
***Cations***						
Copper	Cu	7440-50-8	<LOQ to 9.70	0.021–7	C	[74,122,125,130,142,167,180,181,186,187,188]
Calcium	Ca	7440-70-2	0–7.70	0.30–10.70	C-G	[80,167]
Cadmium	Cd	7440-43-9	<LOQ	0–0.023	C	[51,167]
Chromium	Cr	7440-47-3	-	0	C	[167]
Iron	Fe	7439-89-6	<LOD to 2.20	0–3.60	C-G	[51,80,122,125,142,167]
Potassium	K	7440-09-7	<LOD to 2.20	-	C-G	[80]
Lead	Pb	7439-92-1	<LOD to 0.24	<LOD to 0.19	C	[122,125]
Magnesium	Mg	7439-95-4	0–11.20	0.002–0.210	C-G	[51,80,167]
Manganese	Mn	7439-96-5	-	0.035–2.70	C	[80]
Sodium	Na	7440-23-5	1.90–20	0–36.20	C-G	[80,167]
Nickel	Ni	7440-02-0	-	0–0.017	C	[80]
Strontium	Sr	7440-24-6	<LOQ	-	C	[51]
Cobalt	Co	7440-48-4	-	0	C	[167]
Zinc	Zn	7440-66-6	<LOD to 2.60	0–0.59	C-G	[80,167]

## Data Availability

Not applicable.
